# Engineered virus-like particles for efficient *in vivo* delivery of therapeutic proteins

**DOI:** 10.1016/j.cell.2021.12.021

**Published:** 2022-01-20

**Authors:** Samagya Banskota, Aditya Raguram, Susie Suh, Samuel W. Du, Jessie R. Davis, Elliot H. Choi, Xiao Wang, Sarah C. Nielsen, Gregory A. Newby, Peyton B. Randolph, Mark J. Osborn, Kiran Musunuru, Krzysztof Palczewski, David R. Liu

**Affiliations:** 1 Merkin Institute of Transformative Technologies in Healthcare, Broad Institute of MIT and Harvard, Cambridge, MA, USA; 2 Department of Chemistry and Chemical Biology, Harvard University, Cambridge, MA, USA; 3 Howard Hughes Medical Institute, Harvard University, Cambridge, MA, USA; 4 Gavin Herbert Eye Institute, Center for Translational Vision Research, Department of Ophthalmology, University of California, Irvine, CA, USA; 5 Department of Pharmacology, Case Western Reserve University, Cleveland, OH, USA; 6 Department of Physiology and Biophysics, University of California, Irvine, CA, USA; 7 Cardiovascular Institute, Perelman School of Medicine at the University of Pennsylvania, Philadelphia, PA, USA; 8 Division of Cardiovascular Medicine, Department of Medicine, Perelman School of Medicine at the University of Pennsylvania, Philadelphia, PA, USA; 9 Department of Genetics, Perelman School of Medicine at the University of Pennsylvania, Philadelphia, PA, USA; 10 Department of Pediatrics, Division of Blood and Marrow Transplant and Cellular Therapy, University of Minnesota, Minneapolis, MN, USA; 11 Department of Chemistry, University of California, Irvine, CA, USA; 12 Department of Molecular Biology and Biochemistry, University of California, Irvine, CA, USA

**Keywords:** genome editing, base editing, *in vivo* delivery, ribonucleoproteins, therapeutic gene editing, virus-like particles

## Abstract

Methods to deliver gene editing agents *in vivo* as ribonucleoproteins could offer safety advantages over nucleic acid delivery approaches. We report the development and application of engineered DNA-free virus-like particles (eVLPs) that efficiently package and deliver base editor or Cas9 ribonucleoproteins. By engineering VLPs to overcome cargo packaging, release, and localization bottlenecks, we developed fourth-generation eVLPs that mediate efficient base editing in several primary mouse and human cell types. Using different glycoproteins in eVLPs alters their cellular tropism. Single injections of eVLPs into mice support therapeutic levels of base editing in multiple tissues, reducing serum Pcsk9 levels 78% following 63% liver editing, and partially restoring visual function in a mouse model of genetic blindness. *In vitro* and *in vivo* off-target editing from eVLPs was virtually undetected, an improvement over AAV or plasmid delivery. These results establish eVLPs as promising vehicles for therapeutic macromolecule delivery that combine key advantages of both viral and nonviral delivery.

## Highlights

• Engineered virus-like particles (eVLPs) overcome three bottlenecks to protein delivery
• DNA-free eVLPs efficiently deliver gene editing proteins with minimal off-target editing
• Base editor eVLPs reduced serum Pcsk9 levels 78% following 63% liver editing in mice
• Base editor eVLPs improved visual function in a mouse model of genetic blindness

## Graphical abstract

**Figure:**
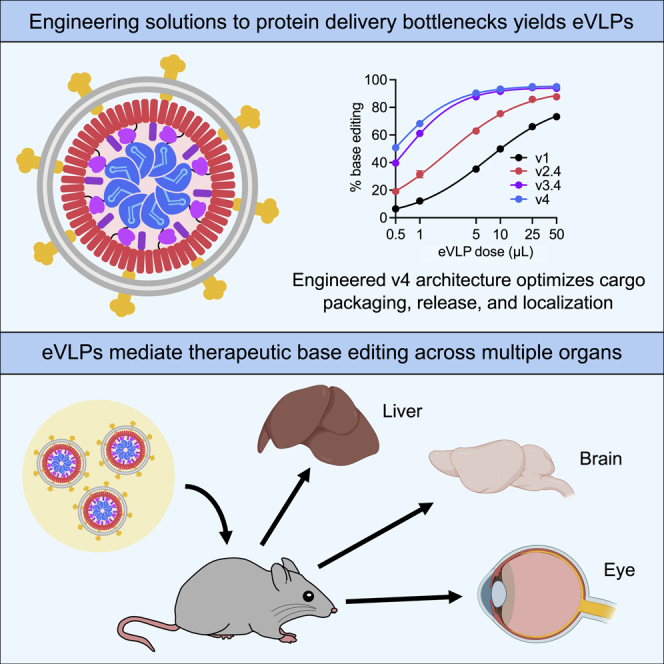

## Teaser

Engineered, DNA-free virus-like particles efficiently deliver gene editing proteins, have minimal off-target effects, can be applied *in vivo* to deliver base editors to multiple organs, and are used to improve visual function in a mouse model of genetic blindness.

## Introduction

Recently developed gene editing agents enable the precise manipulation of genomic DNA in living organisms and raise the possibility of treating the root cause of many genetic diseases (Anzalone et al., 2020; Doudna, 2020; Newby and Liu, 2021). Base editors (BEs) mediate targeted single-nucleotide conversions without requiring double-stranded DNA breaks (DSBs) and thereby minimize undesired consequences of editing such as indels, large deletions (Kosicki et al., 2018; Song et al., 2020), translocations (Giannoukos et al., 2018; Stadtmauer et al., 2020; Turchiano et al., 2021; Webber et al., 2019), chromothripsis (Leibowitz et al., 2021), or other chromosomal abnormalities (Alanis-Lobato et al., 2021). In principle, cytosine base editors (CBEs) (Komor et al., 2016; Nishida et al., 2016) and adenine base editors (ABEs) (Gaudelli et al., 2017) can together correct the majority of known disease-causing single-nucleotide variants (Anzalone et al., 2020; Rees and Liu, 2018). We and others have applied BEs to correct pathogenic point mutations and rescue disease phenotypes in mice and nonhuman primates (Koblan et al., 2021; Levy et al., 2020; Musunuru et al., 2021; Newby and Liu, 2021; Newby et al., 2021; Rothgangl et al., 2021; Suh et al., 2021; Yeh et al., 2020), highlighting the potential of *in vivo* base editing as a therapeutic strategy.

The broad therapeutic application of *in vivo* base editing requires safe and efficient methods for delivering BEs to multiple tissues and organs. The most robust approaches for delivering BEs *in vivo* reported to date involve the use of viruses, such as adeno-associated viruses (AAVs), to deliver BE-encoding DNA to target tissues (Levy et al., 2020; Newby and Liu, 2021). However, viral delivery of DNA-encoding editing agents leads to prolonged expression in transduced cells, which increases the frequency of off-target editing (Akcakaya et al., 2018; Davis et al., 2015; Wang et al., 2020; Yeh et al., 2018). In addition, viral delivery of DNA raises the possibility of viral vector integration into the genome of transduced cells, both of which can promote oncogenesis (Anzalone et al., 2020; Chandler et al., 2017; Koblan et al., 2021). These drawbacks of viral delivery motivate the development of alternative strategies for the *in vivo* delivery of BEs.

An ideal method for delivering gene editing agents *in vivo* would directly deliver proteins or ribonucleoproteins (RNPs) instead of DNA. The short lifetime of RNPs in cells limits opportunities for off-target editing, as demonstrated by previous reports that delivering BE RNPs instead of BE-encoding DNA or mRNA leads to substantially reduced off-target editing, typically without sacrificing on-target editing efficiency (Doman et al., 2020; Newby et al., 2021; Rees et al., 2017). While we and others have previously reported successful base editing in the mouse inner ear and retina following local administration of lipid-encapsulated BE RNPs (Jang et al., 2021; Yeh et al., 2018), no generalizable strategy for delivering BE RNPs to multiple tissues and organs *in vivo* has been reported previously.

Virus-like particles (VLPs), assemblies of viral proteins that can infect cells but lack viral genetic material, have emerged as potentially promising vehicles for delivering gene editing agents as RNPs (Campbell et al., 2019; Choi et al., 2016; Gee et al., 2020; Hamilton et al., 2021; Indikova and Indik, 2020; Lyu et al., 2019, 2021; Mangeot et al., 2019; Yao et al., 2021). VLPs that deliver RNP cargoes exploit the efficiency and tissue targeting advantages of viral delivery but avoid the risks associated with viral genome integration and prolonged expression of the editing agent. However, existing VLP-mediated strategies for delivering gene editing agent RNPs thus far support only modest editing efficiencies with limited validation of therapeutic efficacy *in vivo* (Campbell et al., 2019; Choi et al., 2016; Gee et al., 2020; Hamilton et al., 2021; Indikova and Indik, 2020; Lyu et al., 2019, 2021; Mangeot et al., 2019; Yao et al., 2021). Indeed, to our knowledge, therapeutic levels of postnatal *in vivo* gene editing using RNP-packaging VLPs have not been previously reported.

Here, we describe the development and application of eVLPs, an engineered VLP platform for packaging and delivering both *in vitro* and *in vivo* therapeutic RNPs, including base editors and Cas9 nuclease, thereby offering key advantages of both viral and nonviral delivery strategies. Extensive VLP architecture engineering yielded fourth-generation (v4) eVLPs that package an average of 16-fold more BE RNP compared with initial designs that were based on previously reported VLPs (Mangeot et al., 2019). These v4 eVLPs enable highly efficient base editing with minimal off-target editing in a variety of cell types, including multiple immortalized cell lines, primary human and mouse fibroblasts, and primary human T cells, as well as a 4.7-fold improvement in Cas9 nuclease-mediated indel formation compared with a previously reported Cas9-VLP. Single injections of eVLPs into mice mediated efficient base editing of various target genes in multiple organs including brain, liver, and retina. In the liver, eVLPs strongly knocked down serum Pcsk9 levels, and in the retina eVLPs partially restored visual function in a mouse model of genetic blindness. Our results establish eVLPs as a platform for transiently delivering gene editing agents *in vivo* with therapeutically relevant efficiencies and minimized risk of off-target editing or DNA integration, and may similarly enable the efficient *in vivo* delivery of other proteins and RNPs.

## Results

### A retroviral scaffold supports efficient base editor VLPs

We hypothesized that retroviruses would be an attractive scaffold for engineering base editor VLPs (BE-VLPs). Retroviral capsids generally lack the rigid symmetry requirements of many nonenveloped icosahedral viruses (Zhang et al., 2015), suggesting increased structural flexibility to incorporate nonnative protein cargoes. Additionally, retrovirus tropisms can be modulated by pseudotyping virions with different envelope glycoproteins, which could enable the targeting of BE-VLPs to specific cell types (Cronin et al., 2005). Previous work has demonstrated that fusing a desired protein cargo to the C-terminus of retroviral gag polyproteins is sufficient to the direct packaging of that cargo protein within retroviral particles (Kaczmarczyk et al., 2011; Voelkel et al., 2010). More recently, similar strategies have been applied to package Cas9 RNPs within retroviral particles (Hamilton et al., 2021; Mangeot et al., 2019). Therefore, we sought to investigate whether retroviral scaffolds could support efficient BE-VLP formation in a manner that preserves BE activity.

**Figure 1:**
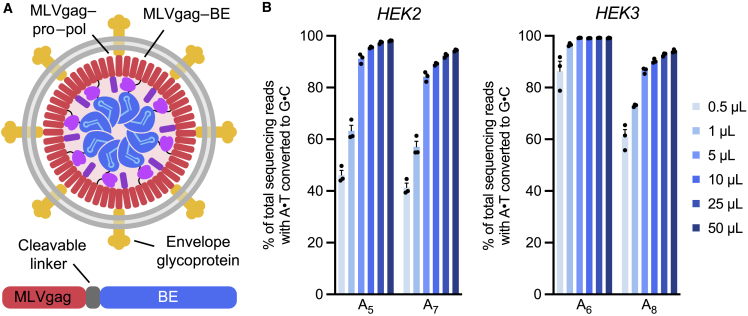
BE-VLP architecture and initial (v1) editing efficiencies (A) Schematic of BE-VLPs. Base-editor (BE) protein is fused to the C-terminus of murine leukemia virus (MLV) gag polyprotein via a linker that is cleaved by the MLV protease upon particle maturation. (B) Adenine base editing efficiencies of v1 BE-VLPs at two genomic loci in HEK293T cells. The protospacer positions of the target adenines are denoted by subscripts (i.e., A_5_, adenine at position 5), where the PAM is positions 21–23. Data are shown as individual data points and mean ± SEM for n = 3 biological replicates.

As an initial (v1) BE-VLP design, we fused ABE8e, a highly active adenine base editor (Richter et al., 2020), to the C-terminus of the Friend murine leukemia virus (FMLV) gag polyprotein via a linker peptide that would be cleaved by the FMLV protease upon particle maturation (Figure 1A). FMLV-based VLPs have been previously used successfully to package and deliver Cas9 RNPs (Mangeot et al., 2019). We produced BE-VLPs by transfecting Gesicle 293T producer cells with plasmids expressing this FMLV gag–ABE8e fusion construct, wild-type FMLV gag–pro–pol polyprotein, the vesicular stomatitis virus G (VSV-G) envelope glycoprotein, and an sgRNA-targeting HEK293T cell genomic site 2 or site 3, hereafter referred to as *HEK2* or *HEK3*.

**Figure S1:**
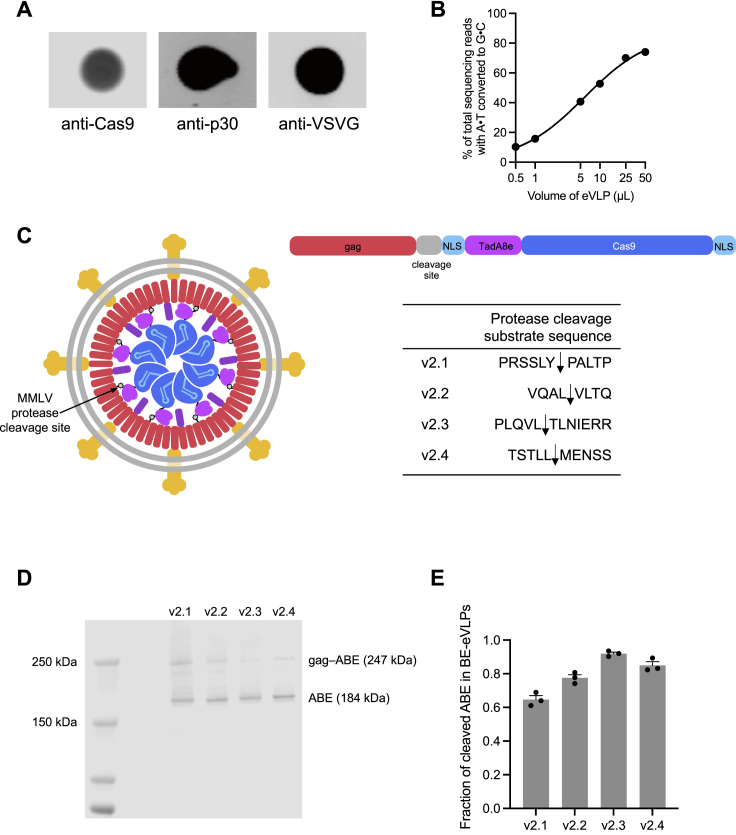
Engineering and characterization of v1 BE-VLPs and v2 BE-eVLPs, related to Figures 1 and 2 (A) Validation of VLP production. Immunoblot analysis of proteins from purified BE-VLPs using anti-Cas9, anti-p30 and anti-VSV-G antibodies. (B) Adenine base editing efficiencies of v1 BE-VLPs at position A_7_ of the *BCL11A* enhancer site in HEK293T cells. Values and error bars reflect mean ± SEM of n = 3 biological replicates. Data were fit to four-parameter logistic curves using nonlinear regression. (C) Schematic of an immature BE-VLP with ABE8e fused to the gag structural protein. Various MMLV protease cleavage sites were inserted between gag and ABE8e to determine the optimal cleavable sequence that promotes liberation of ABE8e from gag during proteolytic virion maturation. Arrows indicate the cleavage site. (D) Representative western blot evaluating cleaved ABE8e versus full-length gag–ABE8e in purified v2 BE-eVLP variants. (E) Densitometry-based quantification of the cleaved ABE8e fraction from western blots. Data are shown as individual data points and mean values ± SEM for n = 3 technical replicates.

After harvesting BE-VLPs from producer cell supernatant, we transduced HEK293T cells *in vitro* with concentrated BE-VLPs. Encouragingly, v1 BE-VLPs robustly edited the *HEK2* and *HEK3* genomic loci with efficiencies >97% at the highest doses in unsorted cells (Figure 1B). We confirmed via immunoblotting that these BE-VLPs contained Cas9, the murine leukemia virus (MLV) capsid, and VSV-G proteins (Figure S1A). These observations indicated that the FMLV retroviral scaffold supports BE-VLP formation and that v1 BE-VLPs can efficiently transduce and edit HEK293T cells *in vitro*.

**Figure S2:**
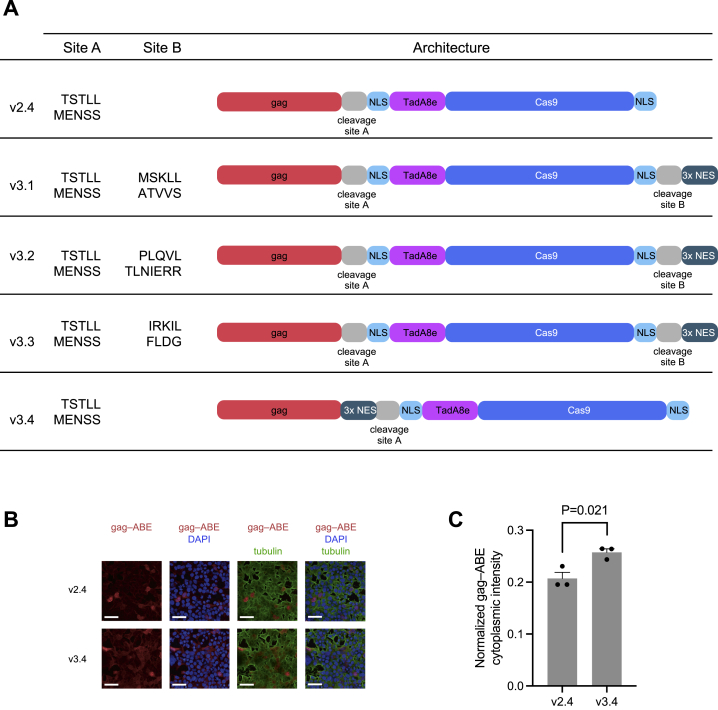
Improving gag–ABE localization in producer cells, related to Figure 2 (A) v2.4 and v3 BE-eVLP constructs. Three HIV NESs were fused to either the C-terminus or N-terminus of the gag–ABE fusion. We incorporated a protease-cleavable linker between ABE and the NES sequences such that the final BE cargo would be devoid of NESs following proteolytic virion maturation. (B) Representative immunofluorescence image of producer cells transfected with the v2.4 gag–ABE construct or the v3.4 gag–3xNES–ABE construct. After 48 h post-transfection, cells were fixed in paraformaldehyde and stained with anti-tubulin antibody (green) to stain the cytoskeleton, DAPI (blue) for nuclei staining and anti-Cas9 antibody (red) to visualize the gag–ABE fusion. Scale bars, 50 μm. (C) Automated image analysis-based quantification of cytoplasmic localization of the v2.4 gag–ABE construct or the v3.4 gag–3xNES–ABE construct. Data are shown as individual data points and mean values ± SEM for n = 3 technical replicates. p values were calculated using a two-sided t test.

### Improving cargo release after VLP maturation

While v1 BE-VLPs robustly edited the *HEK2* and *HEK3* loci in HEK293T cells, these commonly used test loci are especially amenable to gene editing and lack therapeutic relevance (Anzalone et al., 2020). To begin to evaluate the therapeutic potential of BE-VLPs, we assessed their ability to install mutations in the *BCL11A* erythroid-specific enhancer that upregulate the expression of fetal hemoglobin in erythrocytes, an established base editing strategy for the treatment of β-hemoglobinopathies (Richter et al., 2020; Zeng et al., 2020). We observed that v1 BE-VLPs achieved 73% editing at the *BCL11A* enhancer locus in HEK293T cells at high doses, but editing levels dropped steeply with decreasing doses (Figure S1B). These results indicated that v1 BE-VLP activity could be improved.

**Figure 2:**
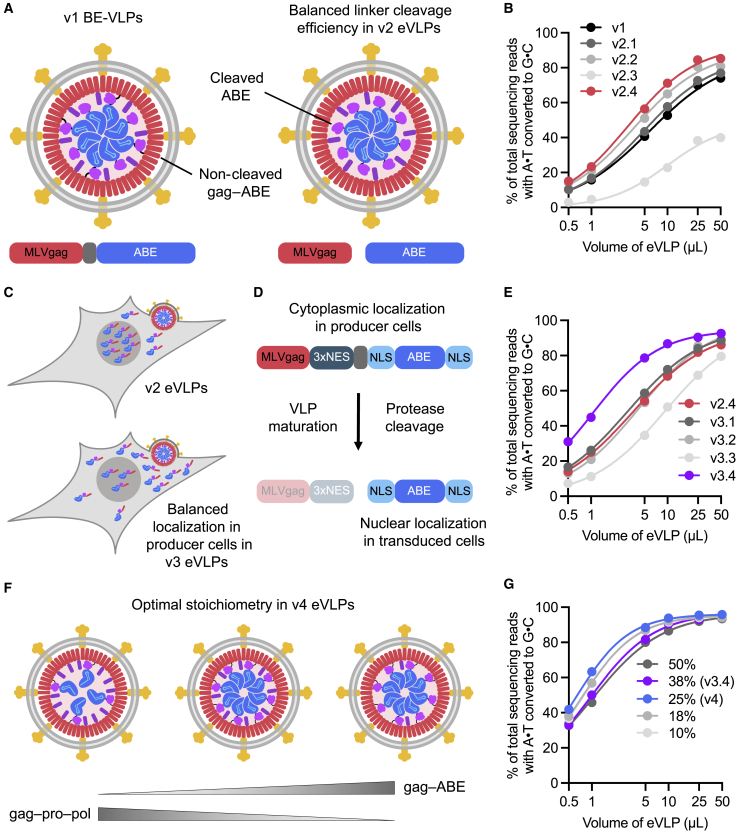
Identifying and engineering solutions to bottlenecks that limit VLP potency results in v2, v3, and v4 eVLPs (A) More efficient linker cleavage leads to improved cargo release after VLP maturation. (B) Adenine base editing efficiencies of v1 and v2 BE-eVLPs at position A_7_ of the *BCL11A* enhancer site in HEK293T cells. (C) Improved localization of cargo in producer cells leads to more efficient incorporation into eVLPs. (D) Installing a 3xNES motif upstream of the cleavable linker encourages cytoplasmic localization of gag–3xNES–cargo in producer cells but nuclear localization of free ABE cargo in transduced cells. (E) Adenine base editing efficiencies of v2.4 and v3 BE-eVLPs at position A_7_ of the *BCL11A* enhancer site in HEK293T cells. (F) The optimal gag–cargo:gag–pro–pol stoichiometry balances the amount of cargo protein per particle with the amount of MMLV protease required for efficient particle maturation. (G) Adenine base editing efficiencies of v3.4 eVLPs with different gag–ABE:gag–pro–pol stoichiometries at position A_7_ of the *BCL11A* enhancer site in HEK293T cells. Legend denotes % gag–ABE plasmid of the total amount of gag–ABE and gag–pro-pol plasmids. (B, E, and G) Values and error bars reflect mean ± SEM of n = 3 biological replicates. Data were fitted to four-parameter logistic curves using nonlinear regression.

Cleavage of the gag–ABE8e linker by the MLV protease after particle maturation is required to liberate free ABE8e RNP. We reasoned that linker-cleavage efficiency might bottleneck BE-VLP editing (Figure 2A). To test this hypothesis, we constructed a series of second-generation (v2) engineered BE-eVLPs that contain a variety of protease-cleavable linker sequences between the MLV gag and ABE8e (Figure S1C). First, we switched the retroviral scaffold from Friend MLV to Moloney MLV (MMLV), a similar MLV strain whose protease-substrate specificity has been extensively characterized (Fehér et al., 2006). We then screened four different linker sequences that were known to be cleaved with varying efficiencies by the MMLV protease and identified several new gag–ABE8e linkers that improved editing efficiencies compared with v1 BE-VLPs (Figure 2B). Specifically, v2.4 BE-eVLPs exhibited 1.2–1.5-fold higher editing efficiencies at all doses tested relative to v1 BE-VLPs (Figure 2B). To investigate the cleavage efficiencies of the linker sequences in v2.1–v2.4 BE-eVLPs, we performed western blots to determine the fraction of cleaved ABE8e versus full-length gag–ABE8e present in purified BE-VLPs. This analysis revealed that the v2.4 linker is cleaved more efficiently than the v2.1 and v2.2 linkers but less efficiently than the v2.3 linker (Figures S1D and S1E).

These results support a model in which the linker sequence in v2.4 BE-eVLPs is cleaved at an optimal rate that supports efficient release of ABE8e RNP after VLP maturation but minimizes premature release of ABE8e RNP prior to its incorporation into VLPs. Our findings demonstrate that the gag–cargo protein linker sequence is an important parameter of VLP architectures and that optimizing this sequence to balance the linker-cleavage kinetics between these two constraints can improve eVLP activity.

### Improving cargo localization and loading into eVLPs

Previously optimized BEs are fused at their N- and C-termini to bipartite nuclear localization signals (NLSs), which promotes nuclear import of BEs and enhances their access to genomic DNA (Koblan et al., 2018). However, gag–BE fusions must be localized to the cytoplasm and outer membrane of producer cells in order to be incorporated into VLPs as they form (Figure 2C). We speculated that the presence of two NLSs within the gag–BE fusion may hamper gag–BE localization to the outer membrane and impede BE incorporation into VLPs.

To encourage cytosolic gag–cargo localization in producer cells, we designed third-generation (v3) eVLP architectures that contain nuclear export signals (NESs) in addition to NLSs. Previous work has demonstrated that MLV-based VLPs can tolerate the addition of NESs at multiple locations within the gag protein (Wu and Roth, 2014). In our v3 designs, we placed MMLV protease-cleavable linker sequences at locations next to NESs to ensure that the NESs would be cleaved from the cargo following VLP maturation (Figures 2D and S2A), thereby liberating NLS-flanked cargo proteins that could be efficiently imported into the nuclei of the transduced cells.

**Figure S3:**
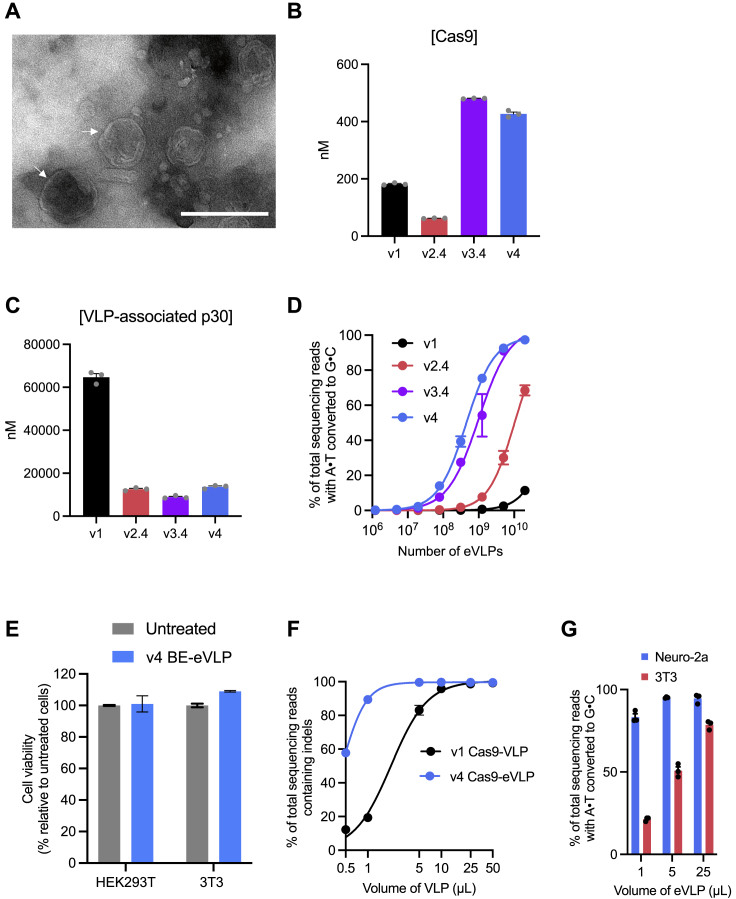
Characterization of BE-eVLPs, related to Figure 3 (A) Representative negative-stain transmission electron micrograph (TEM) of v4 BE-eVLPs. Scale bar, 200 nm. (B and C) Protein content for v1, v2.4, v3.4, and v4 BE-eVLPs was measured by anti-Cas9 or anti-MLV(p30) ELISA. Data are shown as individual data points and mean values ± SEM for n = 3 technical replicates. (D) Comparison of editing efficiencies with particle number-normalized v1, v2.4, v3.4 and v4 BE-VLPs at the *BCL11A* enhancer site in HEK293T cells. Data are shown as mean values ± SEM for n = 3 biological replicates. (E) Cell viability after v4 BE-eVLP treatment of HEK293T cells and NIH 3T3 fibroblasts. Data are shown as mean values ± SEM for n = 3 biological replicates. (F) Indels frequencies generated by v1 Cas9-VLP and v4 Cas9-eVLPs at the *EMX1* locus in HEK293T cells. Data are shown as mean values ± SEM for n = 3 biological replicates. Data were fit to four-parameter logistic curves using nonlinear regression. (G) Adenine base editing efficiencies of VSV-G-pseudotyped v4 BE-eVLPs in Neuro-2a cells or 3T3 fibroblasts. Data are shown as individual data points and mean values ± SEM for n = 3 biological replicates.

All v3 BE-eVLP architectures contained the optimal gag–ABE8e linker sequence from v2.4 BE-eVLPs. BE-eVLPs v3.1, v3.2, and v3.3 harbor a 3xNES motif fused at the C-terminus of ABE8e via an additional MMLV protease-cleavable linker and exhibited comparable or lower efficiencies relative to v2.4 BE-eVLPs (Figure 2E). However, v3.4 BE-eVLPs, which contain a 3xNES motif at the C-terminus of MMLV gag immediately before the v2.4 optimized cleavable linker sequence, exhibited 1.1–2.1-fold improvements in editing efficiencies at the *BCL11A* enhancer locus at all doses tested relative to v2.4 BE-eVLPs (Figure 2E). Notably, v3.4 BE-eVLPs require only a single viral protease cleavage event to liberate NLS-flanked, NES-free BEs (Figures 2D and S2A), compared with the two distinct cleavage events required in v3.1, v3.2, and v3.3 BE-eVLPs, which might explain their superior efficiency. To further investigate the effect of NES addition on gag–ABE localization, we performed immunofluorescence microscopy of producer cells transfected with the v3.4 gag–3xNES–ABE construct or the v2.4 gag–ABE construct. This analysis revealed a 1.3-fold increase in cytoplasmic localization of ABE protein detected in v3.4-transfected producer cells relative to v2.4-transfected producer cells (Figures S3B and S3C). These results demonstrate that BE-eVLP activity can be improved by promoting the extranuclear localization of the gag–BE fusion in producer cells while maintaining the nuclear localization of the BEs released into transduced cells.

### Improving component stoichiometry of eVLPs

Finally, we optimized the gag–cargo:gag–pro–pol stoichiometry of v3.4 eVLPs. We hypothesized that an optimal gag–cargo:gag–pro–pol stoichiometry would balance the amount of gag–cargo available to be packaged into VLPs with the amount of MMLV protease (“pro” in gag–pro–pol) required for VLP maturation (Figure 2F). To modulate this stoichiometry, we varied the ratio of gag–3xNES–ABE8e to wild-type MMLV gag–pro–pol plasmids transfected for VLP production. We found that increasing the amount of gag–BE plasmid beyond the original proportion used for producing v3.4 BE-eVLPs (38% gag–BE plasmid and 62% gag–pro–pol plasmid) did not improve editing efficiencies (Figure 2G). Decreasing the proportion of gag–BE plasmid from 38% to 25% modestly improved editing efficiencies (Figure 2G). However, further decreasing the proportion of gag–BE plasmid below 25% reduced editing efficiencies (Figure 2G).

The results of this final round of VLP engineering revealed a fourth-generation (v4) BE-eVLP formulation (Figure 2G) which combines the optimal gag–BE:gag–pro–pol stoichiometry (25% gag–BE) with the v3.4 BE-eVLP architecture. We visualized v4 BE-eVLPs by transmission electron microscopy and confirmed their spherical morphology and approximate particle diameter of 100–150 nm (Figure S3A).

**Figure 3:**
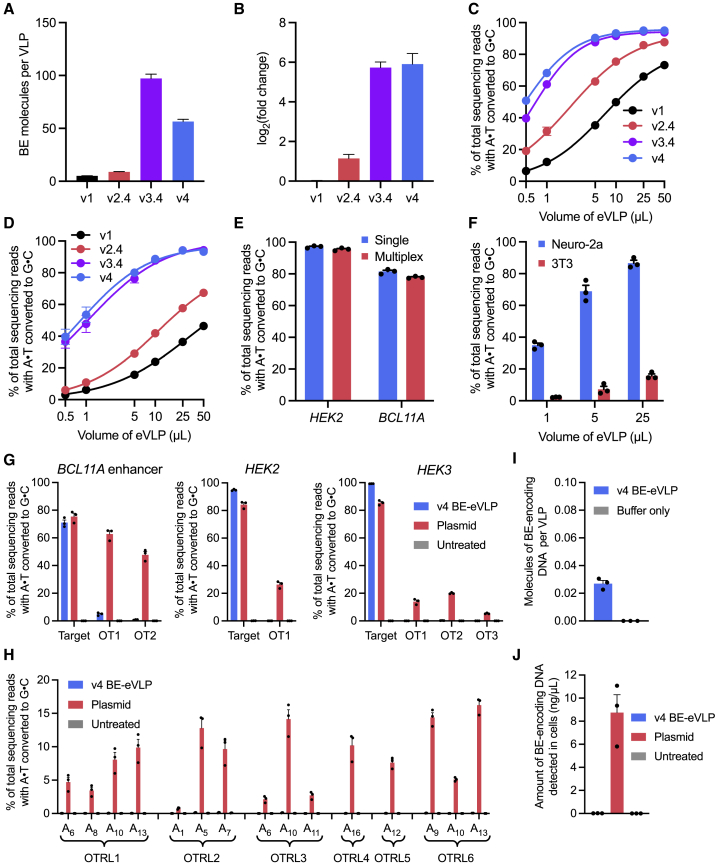
Characterization of BE-eVLPs (A) Quantification of BE molecules per eVLP by anti-Cas9 and anti-MLV (p30) ELISA. Values and error bars reflect mean ± SEM of n = 3 replicates. (B) Quantification of relative sgRNA abundance by RT-qPCR using sgRNA-specific primers, normalized relative to v1 sgRNA abundance. Values and error bars reflect mean ± SEM of n = 3 technical replicates. (C and D) Comparison of editing efficiencies with v1, v2.4, v3.4, and v4 BE-eVLPs at the *BCL11A* enhancer site in HEK293T cells (C) and at the *Dnmt1* site in NIH 3T3 cells (D). Values and error bars reflect mean ± SEM of n = 3 biological replicates. Data were fitted to four-parameter logistic curves using nonlinear regression. (E) Adenine base editing efficiencies in HEK293T cells of either single v4 BE-eVLPs targeting the *HEK2* or *BCL11A* enhancer loci separately, or multiplex v4 BE-eVLPs targeting both loci simultaneously. (F) Adenine base editing efficiencies of FuG-B2-pseudotyped v4 BE-eVLPs at the *Dnmt1* locus in Neuro-2a cells or 3T3 fibroblasts. (G) Adenine base editing efficiencies at three on-target genomic loci and their corresponding Cas-dependent off-target sites in HEK293T cells treated with v4 BE-eVLPs or ABE8e plasmid. OT1, off-target site 1; OT2, off-target site 2; OT3, off-target site 3. (H) Cas-independent off-target editing frequencies at six off-target R-loops in HEK293T cells treated with v4 BE-eVLPs or ABE8e plasmid. OTRL, off-target R-loop. See also Figure S4A for the experimental timeline and Figure S4B for on-target editing controls. (I) Molecules of BE-encoding DNA per v4 BE-eVLP detected by qPCR of lysed eVLPs or lysis buffer only. (J) Amount of BE-encoding DNA detected by qPCR of lysate from HEK293T cells that were either treated with v4 BE-eVLPs or transfected with BE-encoding plasmids. (E–J) Data are shown as individual data points and mean ± SEM for n = 3 biological replicates.

Next, we determined the effects of our architecture engineering on the protein content of BE-eVLPs. We performed anti-Cas9 and anti-MLV(p30) ELISAs to quantify the number of BE molecules and p30 (MLV capsid) molecules present in v1 through v4 BE-eVLPs (Figures S3B and S3C). These experiments revealed that v2.4, v3.4, and v4 BE-eVLPs contain 1.8-, 19.2-, and 11-fold more BE cargo protein molecules per particle, respectively, compared with v1 BE-VLPs (Figure 3A). This increase in BE protein content per particle correlates with an increase in the relative amount of sgRNAs per particle as measured by targeted RT-qPCR of lysed VLPs (Figure 3B). Interestingly, v4 BE-eVLPs contain fewer BE protein molecules per particle than v3.4 BE-eVLPs but the same amount of sgRNA molecules, which suggests that v3.4 and v4 BE-eVLPs may contain similar amounts of active BE RNPs per particle. Additionally, v4 BE-eVLPs are produced at higher titer than v3.4 BE-eVLPs (Figure S3C).

These results support a model in which increasing the number of active BE RNP molecules per particle can improve BE-eVLP editing efficiencies. However, increasing the number of BE molecules per particle beyond a certain threshold can be harmful, since these additional BE molecules do not appear to be complexed with sgRNAs, and there is an apparent trade-off between the number of cargo molecules incorporated per VLP and overall VLP titers. Together, these results reveal additional important parameters that influence eVLP efficiencies and demonstrate how these parameters can be improved by modulating gag–cargo localization and gag–cargo:gag–pro–pol stoichiometry.

### v4 eVLPs support potent, high-efficiency gene editing

The successive VLP engineering efforts described above substantially improved editing efficiencies of v4 BE-eVLPs at the *BCL11A* enhancer locus in HEK293T cells to 95% at the maximal dose (Figure 3C); v4 BE-eVLPs exhibit a 5.6-fold improvement in editing efficiency per unit volume compared with v1 BE-VLPs and a 2.2-fold improvement compared with v2.4 BE-eVLPs (Figure 3C). We also observed that v4 BE-eVLPs exhibit 8.5-fold improvements in base editing activity per viral particle in HEK293T cells (Figure S3D). To confirm that v4 VLP engineering supports general base editing improvements that are not restricted to one particular genomic locus or cell line, we tested v1, v2.4, v3.4, and v4 BE-eVLPs targeting the *Dnmt1* locus in 3T3 mouse fibroblasts. We observed a very similar trend in the editing efficiencies of the four eVLP architectures, with an 8.6-fold improvement in editing efficiency per unit volume of v4 BE-eVLPs compared with v1 BE-VLPs in 3T3 cells (Figure 3D). Additionally, treatment with v4 BE-eVLPs had no negative impact on the viability of HEK293T or 3T3 cells (Figure S3E). v4 BE-eVLPs also supported robust multiplex editing of the *BCL11A* enhancer and *HEK2* genomic loci in HEK293T cells (Figure 3E). These results show that v4 BE-eVLPs mediate high-efficiency base editing with minimal impact to the viability of treated cells.

We hypothesized that the engineered v4 eVLP architecture might similarly improve VLP-mediated delivery of other proteins in addition to BEs. To test this possibility, we constructed v1 and v4 VLPs that packaged Cas9 nuclease (Cas9-VLPs) and an sgRNA targeting the *EMX1* genomic locus. We observed a 4.7-fold improvement in indel frequencies per unit volume generated by v4 Cas9-eVLPs compared with v1 Cas9-VLPs in HEK293T cells (Figure S3F). This observation suggests that the engineered v4 eVLP architecture offers improvements to VLP-mediated delivery of proteins that are not limited to BEs.

The cellular tropism of VLPs can be modulated by producing them with different envelope glycoproteins, as was previously used to modulate the tropism of Cas9-VLPs (Hamilton et al., 2021). To investigate whether BE-eVLPs can be programmed to target certain cell types, we produced v4 BE-eVLPs pseudotyped with the FuG-B2 envelope glycoprotein (Kato et al., 2011). FuG-B2 is an engineered envelope glycoprotein that contains the extracellular and transmembrane domains of the rabies virus envelope glycoprotein and the cytoplasmic domain of VSV-G and can be used to pseudotype lentiviruses for neuron-specific transduction (Kato et al., 2011). Indeed, we observed that FuG-B2-pseudotyped v4 BE-eVLPs efficiently transduce and edit Neuro-2a cells (a mouse neuroblastoma cell line) but not mouse 3T3 fibroblasts (Figures 3F and S3G). These results validate that the tissue specificity of eVLPs can be targeted by swapping in other glycoproteins such as those used to pseudotype lentiviruses to transduce specific cell populations.

Collectively, these findings identify factors that influence VLP activity, and demonstrate that extensively engineering the protease-cleavable linker sequence, gag–cargo localization, and gag–cargo:gag–pro–pol stoichiometry can overcome bottlenecks that limit VLP potency. These results also establish v4 BE-eVLPs as a robust method for delivering BE RNPs in cultured cells.

### v4 BE-eVLPs show minimal off-target editing or DNA integration

Given that v4 BE-eVLPs exhibit robust on-target base editing at several endogenous genomic loci in multiple cell types, we next sought to assess their off-target editing profiles. BEs can mediate Cas-dependent off-target editing at a subset of Cas9 off-target binding sites, as well as Cas-independent off-target editing at a low level throughout the genome (Anzalone et al., 2020). To evaluate Cas-dependent off-target editing by v4 BE-eVLPs relative to ABE8e plasmid transfection in HEK293T cells, we performed targeted amplicon sequencing of known Cas9 off-target sites associated with three different sgRNAs targeting the *HEK2*, *HEK3*, and *BCL11A* enhancer loci. We observed comparable or higher on-target editing efficiency from v4 BE-eVLPs compared with plasmid transfection at these three genomic loci, but 12- to 900-fold lower Cas-dependent off-target editing from v4 BE-eVLPs (Figure 3G).

**Figure S4:**
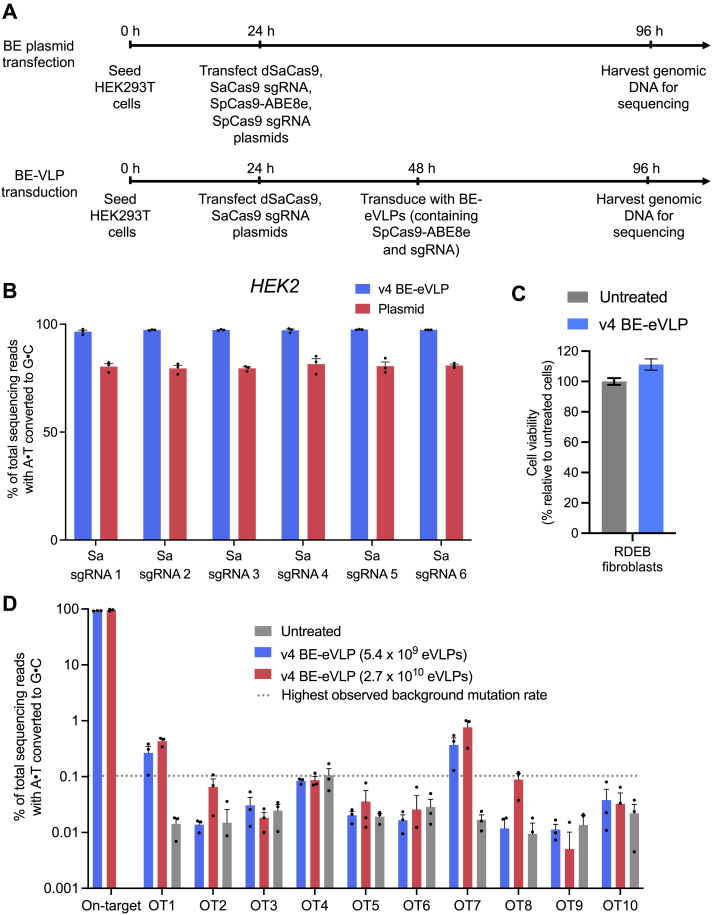
Off-target editing by v4 BE-eVLPs, related to Figures 3 and 4 (A) Experimental timeline for the orthogonal R-loop assay. (B) On-target editing controls for the orthogonal R-loop experiment. Data are shown as individual data points and mean values ± SEM for n = 3 biological replicates. (C) Cell viability following v4 BE-eVLP treatment of RDEB fibroblasts. Data are shown as mean values ± SEM for n = 3 biological replicates. (D) DNA sequencing reads containing A⋅T-to-G⋅C mutations within protospacer positions 4–10 for ten previously identified off-target loci from the genomic DNA of v4-BE-eVLP-treated RDEB patient-derived fibroblasts. The dotted gray line represents the highest observed background mutation rate of 0.1%. Data are shown as individual data points and mean values ± SEM for n = 3 biological replicates.

To evaluate Cas-independent off-target DNA editing, we performed an orthogonal R-loop assay, which multiple labs have previously validated as a strategy for assessing the ability of a BEs to deaminate DNA in an unguided manner without requiring whole-genome sequencing (Doman et al., 2020; Yu et al., 2020). Compared with transfection of DNA plasmid encoding the same BE, v4 BE-eVLPs exhibited a >100-fold reduction in Cas-independent off-target editing, down to virtually undetected levels (Figures 3H and S4B). These results confirm and extend previous findings that off-target editing by highly active BEs can be substantially minimized with RNP delivery (Doman et al., 2020; Jang et al., 2021; Lyu et al., 2021; Newby et al., 2021; Rees and Liu, 2018; Richter et al., 2020; Yeh et al., 2018) and highlight the ability of eVLPs to support highly efficient on-target base editing with minimal off-target editing.

In principle, the DNA-free nature of eVLPs avoids the possibility of DNA integration into the genomes of transduced cells, an important safety advantage over existing viral delivery modalities (David and Doherty, 2017; Milone and O’Doherty, 2018). We verified by qPCR that purified v4 BE-eVLPs contain <0.03 molecules of BE-encoding DNA per VLP (Figure 3I). Additionally, while we detected substantial amounts (8.7 ng/μL) of BE-encoding DNA in cellular lysate from HEK293T cells that were transfected with BE-encoding plasmids, we did not detect BE-encoding DNA in cellular lysate from v4 BE-eVLP-treated HEK293T cells above background levels in samples from untreated cells (<0.02 ng/μL) (Figure 3J). These results demonstrate that BE-eVLPs do not expose transduced cells to detected levels of DNA-encoding BEs, thereby minimizing the possibility of genomic integration of cargo DNA.

### v4 BE-eVLPs efficiently edit primary human and mouse cells

**Figure 4:**
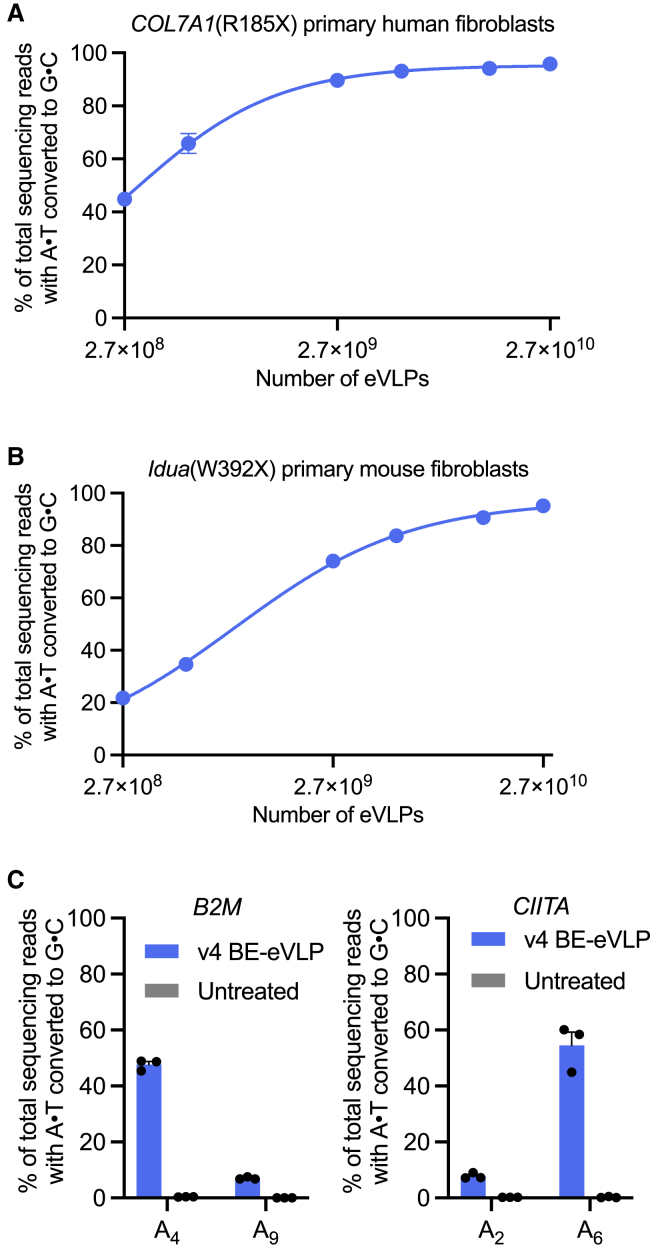
Base editing in primary human and mouse cells using v4 BE-eVLPs (A) Correction efficiencies of the *COL7A1*(R185X) mutation in patient-derived primary human fibroblasts. (B) Correction efficiencies of the *Idua*(W392X) mutation in primary mouse fibroblasts. (A and B) Values and error bars reflect mean ± SEM of n = 3 biological replicates. Data were fitted to four-parameter logistic curves using nonlinear regression. (C) Adenine base editing efficiencies at the *B2M* and *CIITA* loci in primary human T cells. Data are shown as individual data points and mean ± SEM for n = 3 biological replicates.

To further explore the utility of v4 BE-eVLPs, we assessed their ability to target and edit a variety of primary human or mouse cells *ex vivo*. We previously demonstrated ABE-mediated correction of nonsense mutations in *COL7A1* that cause recessive dystrophic epidermolysis bullosa (RDEB) in primary human patient-derived fibroblasts (Osborn et al., 2020). After transducing primary fibroblasts harboring a homozygous *COL7A1*(R185X) mutation with v4 BE-eVLPs, we observed >95% editing at the target adenine base with no difference in the cellular viability between eVLP-treated and untreated cells (Figures 4A and S4C). Additionally, we observed minimal Cas-dependent off-target editing at 10 previously identified off-target sites (Osborn et al., 2020) (Figure S4D). We also assessed the ability of v4 BE-eVLPs to correct a nonsense mutation in primary fibroblasts derived from a mouse model of mucopolysaccharidosis type IH (Wang et al., 2010). Again, we observed >95% correction of the *Idua*(W392X) mutation following v4 BE-eVLP transduction (Figure 4B). These results validate that BE-eVLP activity is not restricted to immortalized cell lines and demonstrate that v4 BE-eVLPs can achieve levels of base editing in primary human and mouse fibroblasts approaching 100%.

Next, we investigated BE-eVLP-mediated editing in primary human T cells. Gene editing strategies that reduce the expression of immunomodulatory proteins on the surface of T cells, including MHC class I and MHC class II, could advance T-cell therapies by enabling “off-the-shelf” allogeneic chimeric antigen receptor (CAR) T cells. Previous reports have shown that disrupting splice sites in the *B2M* and *CIITA* genes reduces expression of MHC class I and MHC class II in primary human T cells (Gaudelli et al., 2020; LeibundGut-Landmann et al., 2004; Serreze et al., 1994). Treating primary human T cells with v4 BE-eVLPs led to a 45%–60% disruption of *B2M* and *CIITA* splice sites (Figure 4C). Collectively, these results confirm that BE-eVLPs can efficiently edit clinically relevant primary human cell types *ex vivo* and lay a foundation for the further optimization of BE-eVLP editing efficiencies in primary human T cells.

### *In vivo* base editing in the CNS with eVLPs

The robust activity of eVLPs *ex vivo* suggested that they might be promising vehicles for delivering BE RNPs *in vivo*. To begin to assess their *in vivo* delivery efficacy, we first investigated the ability of eVLPs to enable base editing within the mouse central nervous system (CNS). We produced v4 BE-eVLPs that install a silent mutation in mouse *Dnmt1*, a genomic locus known to be amenable to nuclease-mediated indel formation and adenine base editing *in vivo* (Levy et al., 2020; Swiech et al., 2015). To deliver BE-eVLPs to the CNS, we performed neonatal cerebroventricular (P0 ICV) injections, which are direct injections into cerebrospinal fluid that bypass the blood-brain barrier, similar to the intrathecal injections currently used to deliver nusinersen in patients with spinal muscular atrophy (Mercuri et al., 2018).

**Figure 5:**
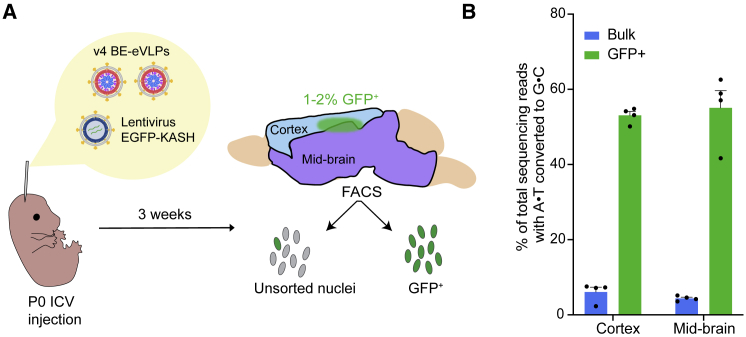
*In vivo* base editing in the central nervous system using v4 BE-eVLPs (A) Schematic of P0 ICV injections of v4 BE-eVLPs. *Dnmt1*-targeting v4 BE-eVLPs were co-injected with a lentivirus encoding EGFP-KASH. Tissue was harvested 3 weeks post-injection, and cortex and mid-brain were separated. Nuclei were dissociated for each tissue and analyzed by high-throughput sequencing as bulk unsorted (all nuclei) or GFP+ nuclei. (B) Adenine base editing efficiencies at the *Dnmt1* locus in bulk unsorted (all nuclei) and GFP+ populations. Data are shown as individual data points and mean ± SEM for n = 4 mice.

We co-injected v4 BE-eVLPs into each hemisphere together with a VSV-G-pseudotyped lentivirus encoding EGFP fused to a nuclear membrane-localized Klarsicht/ANC-1/Syne-1 homology (KASH) domain (Figure 5A). We reasoned that this strategy would enable the isolation of GFP-positive nuclei as a way to enrich cells that were exposed to eVLPs. This approach is particularly useful to determine editing efficiencies following injection in the brain, where many cells may not be accessible. Three weeks post-injection, we analyzed bulk unsorted and GFP-positive nuclei from cortical and mid-brain tissues and assessed base editing by high-throughput sequencing (Figure 5A).

**Figure S5:**
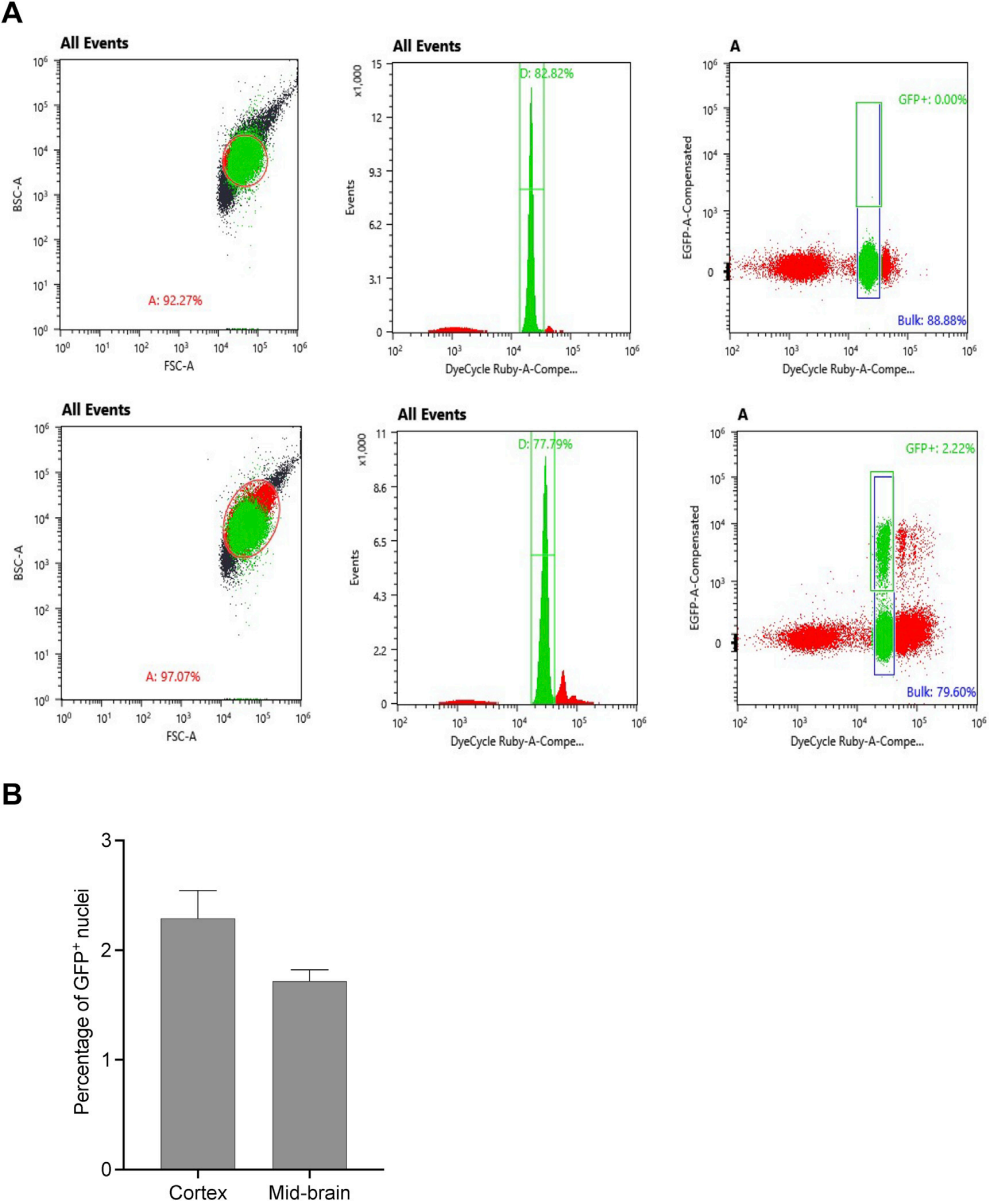
Flow cytometry analysis for nuclei sorting from the mouse brain after P0 ICV injection, related to Figure 5 (A) Singlet nuclei were gated based on FSC/BSC ratio and DyeCycle Ruby signal. The first row demonstrates the gating strategy on a GFP-negative sample. Bulk nuclei correspond to events that passed gate D for singlet nuclei. (B) Percentage of GFP-positive nuclei measured by flow cytometry following P0 ICV injection. Data are shown as mean values + SEM for n = 3 biological replicates.

The frequencies of GFP-positive nuclei in both cortical and mid-brain tissues were low (Figure S5B), consistent with previous reports that the cells transduced by VSV-G-pseudotyped lentiviruses injected into the mouse brain are localized near the injection site (Humbel et al., 2021; Parr-Brownlie et al., 2015), possibly because the size of the viral particles, which have an average diameter of ∼3-fold larger than the width of the brain extracellular space (Thorne and Nicholson, 2006), may hinder diffusion through bulk brain tissue. Encouragingly, we observed 53% and 55% editing in GFP-positive cortex and mid-brain cells, respectively, corresponding to 6.1% and 4.4% editing of bulk cortex and mid-brain (Figure 5B). These data establish BE-eVLPs as a new nonviral delivery system for CNS base editing applications that deliver robust levels of active BE RNP per transduction event, although improvements in transduction efficiency are needed to achieve high levels of editing in bulk brain tissue.

### *In vivo* liver base editing with eVLPs leads to efficient knockdown of Pcsk9

To further explore the utility of BE-eVLPs *in vivo*, we investigated their ability to mediate therapeutic base editing in adult animals. First, we targeted proprotein convertase subtilisin/kexin type 9 (*Pcsk9*), a therapeutically relevant gene involved in cholesterol homeostasis (Abifadel et al., 2003; Fitzgerald et al., 2014). Loss-of-function *PCSK9* mutations occur naturally without apparent adverse health consequences (Abifadel et al., 2003; Cohen et al., 2005, 2006; Hooper et al., 2007; Rao et al., 2018). These individuals have lower levels of low-density lipoprotein (LDL) cholesterol in the blood and a reduced risk of atherosclerotic cardiovascular disease, suggesting that disrupting the *PCSK9* gene could be a promising strategy for the treatment of familial hypercholesterolemia (Musunuru et al., 2021; Rothgangl et al., 2021).

**Figure 6:**
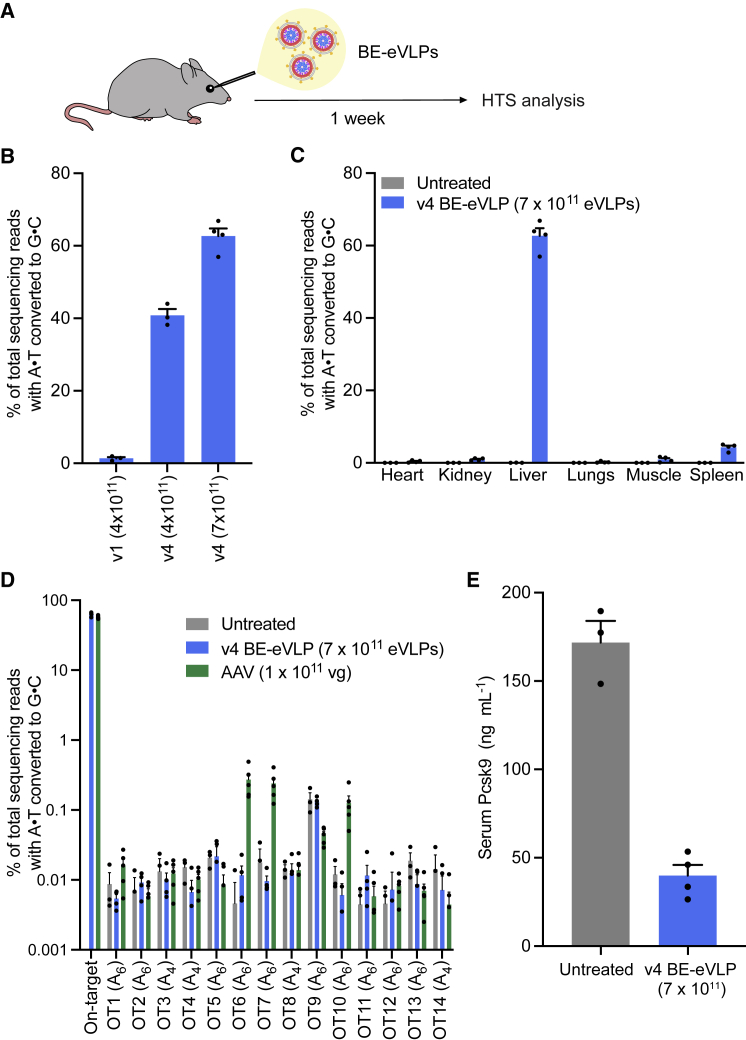
*In vivo* knockdown of Pcsk9 from a single systemic injection of v4 BE-eVLPs (A) Schematic of systemic injections of BE-eVLPs. *Pcsk9*-targeting BE-eVLPs were injected retro-orbitally into 6- to 7-week-old C57BL/6J mice. Organs were harvested one week after injection and the genomic DNA of unsorted cells was sequenced. (B) Adenine base editing efficiencies at the *Pcsk9* exon 1 splice donor in the mouse liver after systemic injection of v1 BE-VLPs or v4 BE-eVLPs. Data are shown as individual data points and mean ± SEM for n = 3 mice (v1 BE-VLP and v4 BE-eVLP at 4 × 10^11^ VLPs) or n = 4 mice (v4 BE-eVLP at 7 × 10^11^ eVLPs). (C) Adenine base editing efficiencies at the *Pcsk9* exon 1 splice donor in the mouse heart, kidney, liver, lungs, muscle, and spleen after systemic injection of 7 × 10^11^ v4 BE-eVLPs. Data are shown as individual data points and mean ± SEM for n = 4 mice (treated) or n = 3 mice (untreated). (D) DNA sequencing reads containing A⋅T-to-G⋅C mutations within protospacer positions 4–10 for the 14 CIRCLE-seq-nominated off-target loci from the livers of v4 BE-eVLP-treated, AAV-treated, and untreated mice. Data are shown as individual data points and mean ± SEM for n = 4 mice (BE-eVLP), n = 5 mice (AAV), or n = 3 mice (untreated). vg, viral genomes. (E) Serum Pcsk9 levels as measured by ELISA. Data are shown as individual data points and mean ± SEM for n = 4 mice (treated) or n = 3 mice (untreated).

We designed and produced v4 BE-eVLPs that target and disrupt the splice donor at the boundary of *Pcsk9* exon 1 and intron 1, a previously established base editing strategy for *Pcsk9* knockdown in the mouse liver (Musunuru et al., 2021; Rothgangl et al., 2021). We performed systemic (retro-orbital) injections of the eVLPs into 6- to 7-week-old adult C57BL/6J mice and measured base editing in the liver one week after injection (Figure 6A). We observed 63% editing in bulk liver following treatment with the highest dose (7 × 10^11^ eVLPs) of v4 BE-eVLPs (Figure 6B), comparable to editing efficiencies typically achieved at this site with optimized, state-of-the-art AAV-based delivery or lipid nanoparticle (LNP)-based mRNA delivery systems (Musunuru et al., 2021; Rothgangl et al., 2021). The engineered v4 BE-eVLP architecture supported 26-fold higher editing levels in the liver than the VLP architecture based on a previously reported design (v1 BE-VLP) administered the same way at the same dose (Figure 6B). These results establish efficient base editing by RNPs at a therapeutically relevant locus in the mouse liver, and show that our engineering of VLPs greatly increased their *in vivo* delivery efficacy.

In mice treated with the highest dose of v4 BE-eVLPs, we also assessed base editing efficiencies in nonliver tissues, including the heart, skeletal muscle, lungs, kidney, and spleen. We observed 4.3% base editing in the spleen, and minimal editing above background levels in the lungs, kidneys, heart, and muscle (Figure 6C). This pattern of editing across tissues is consistent with the previously characterized tissue tropism of intravenously administered VSV-G-pseudotyped particles (Pan et al., 2002).

To assess whether treatment with BE-eVLPs resulted in Cas-dependent off-target editing in liver tissue, we performed CIRCLE-seq (Tsai et al., 2017) to nominate potential off-target loci. From the nominated loci, we selected 14 candidate off-target sites to examine by targeted high-throughput sequencing based on homology near the PAM-proximal region of the protospacer. We observed no detectable off-target editing above background levels at any of these loci in genomic DNA isolated from livers of mice treated with 7 × 10^11^ v4 BE-eVLPs (Figure 6D). In contrast, we observed low but detectable (0.1%–0.3%) levels of off-target editing at three of these loci in genomic DNA isolated from livers of mice treated with dual AAV8 vectors (1 × 10^11^ viral genomes) encoding ABE8e and the same *Pcsk9*-targeting sgRNA (Figure 6D). These results demonstrate that v4 BE-eVLPs can offer comparable on-target editing but minimal off-target editing *in vivo*, an improvement compared to existing viral delivery approaches.

**Figure S6:**
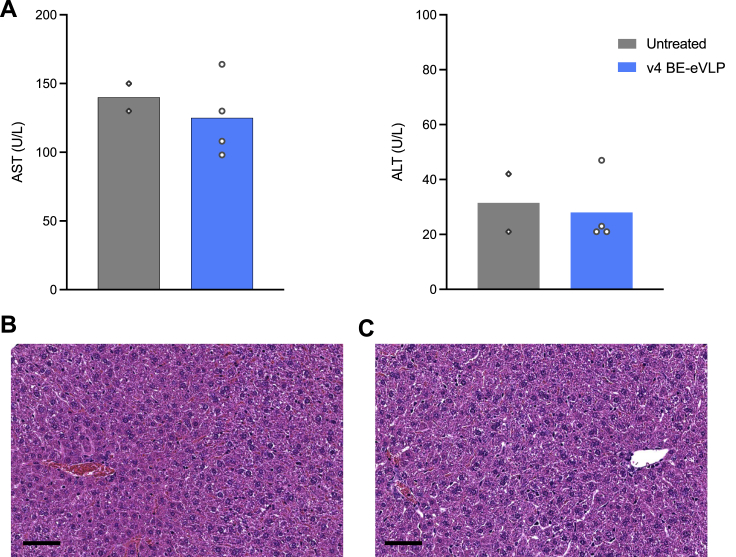
Assessment of liver toxicity following systemic v4 BE-eVLP injection, related to Figure 6 (A) Plasma aspartate transaminase (AST) and alanine transaminase (ALT) levels one week after v4 BE-eVLP injection. (B and C) Histopathological assessment by hematoxylin and eosin staining of livers at 1 week post-injection of (B) untreated mice and (C) v4 BE-eVLP-treated mice. A representative example of each is shown. Scale bars, 50 μm.

Phenotypic analyses performed 1 week post-injection revealed a 78% reduction in serum Pcsk9 protein levels in mice treated with 7 × 10^11^ v4 BE-eVLPs compared to untreated mice (Figure 6E). To assess the potential toxicity of systemically administered eVLPs, we evaluated serum alanine aminotransferase (ALT) and aspartate transaminase (AST) levels, important biomarkers of hepatocellular injury (Meunier and Larrey, 2019), 1 week after injection of 7 × 10^11^ v4 BE-eVLPs. All mice exhibited AST and ALT levels within the normal ranges, and there were no discernible differences between the untreated mice and the eVLP-treated mice (Figure S6A). Additionally, we performed liver histology on samples from eVLP-treated and untreated mice and found no evident morphological differences due to eVLP treatment (Figures S6B and S6C). Together, these results demonstrate that v4 BE-eVLPs can mediate efficient, therapeutically relevant base editing in the mouse liver with no apparent adverse consequences and no detected off-target editing.

### v4 BE-eVLPs restore visual function in a mouse model of genetic blindness

Finally, we applied BE-eVLPs to correct a disease-causing point mutation in an adult mouse model of a genetic retinal disorder. Loss-of-function mutations in multiple genes are associated with various forms of Leber congenital amaurosis (LCA), a family of monogenic retinal disorders that involve retinal degeneration, early-onset visual impairment, and eventual blindness (Cideciyan, 2010; den Hollander et al., 2008). Gene editing approaches hold promise to treat and cure congenital blindness; an ongoing clinical trial (NCT03872479) uses AAV-delivered Cas9 nucleases to disrupt an aberrant splice site in *CEP290* that is associated with rare Leber congenital amaurosis 10 (LCA10). Loss-of-function mutations in other genes, including the retinoid isomerohydrolase *RPE65*, are also candidates for *in vivo* correction using precision gene editing agents (Sodi et al., 2021; Suh et al., 2021).

We investigated whether v4 BE-eVLPs can restore visual function in a mouse model of LCA. The *rd12* mouse model harbors a nonsense mutation in exon 3 of *Rpe65* (c.130C > T; p.R44X) that causes a near-complete loss of visual function (Pang et al., 2005; Suh et al., 2021). A homologous mutation responsible for LCA has recently been identified in people (Zhong et al., 2019), highlighting the clinical relevance of the *rd12* model.

**Figure 7:**
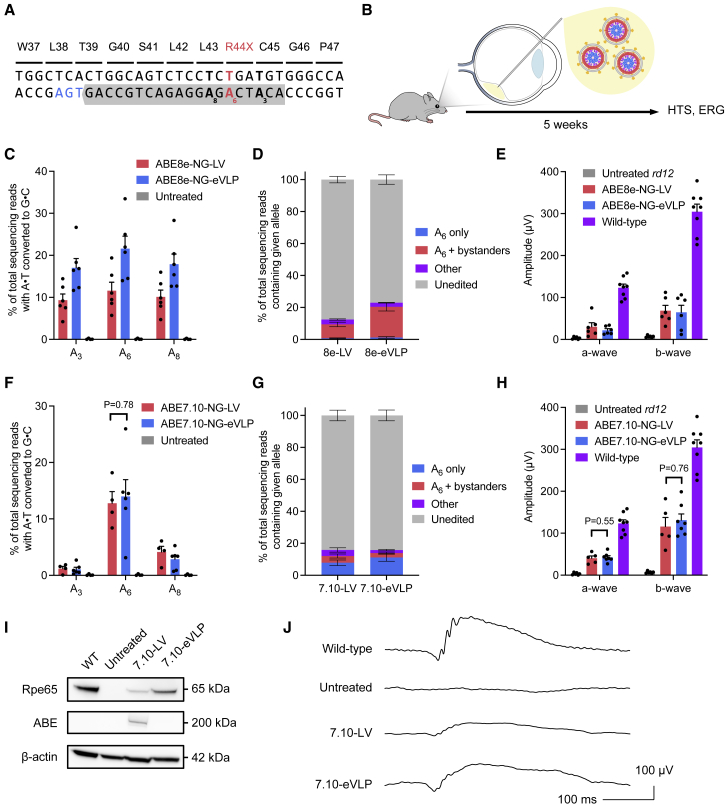
*In vivo* base editing by v4 BE-eVLPs in a mouse model of genetic blindness (A) Schematic of *Rpe65* exon 3 surrounding the R44X mutation (in red), which can be corrected by an A⋅T-to-G⋅C conversion at position A_6_ in the protospacer (shaded gray, PAM in blue). (B) Schematic of subretinal injections. Five weeks post-injection, phenotypic rescue was assessed via ERG and tissues were subsequently harvested for sequencing. HTS, high-throughput sequencing; ERG, electroretinography. (C) Adenine base editing efficiencies at positions A_3_, A_6_, and A_8_ of the protospacer in genomic DNA harvested from *rd12* mice. Data are shown as individual data points and mean ± SEM for n = 6 mice (both treated groups) or n = 4 mice (untreated). (D) Allele frequency distributions of genomic DNA harvested from treated *rd12* mice. Data are shown as mean ± SEM for n = 6 mice. 8e-LV, ABE8e-NG-LV; 8e-eVLP, v4 ABE8e-NG-eVLP. (E) Scotopic a- and b-wave amplitudes measured by ERG following overnight dark adaptation. Data are shown as individual data points and mean ± SEM for n = 8 mice (wild-type), n = 6 mice (ABE8e-NG-LV and v4 ABE8e-NG-eVLP) or n = 4 mice (untreated). (F) Adenine base editing efficiencies at positions A_3_, A_6_, and A_8_ of the protospacer in genomic DNA harvested from *rd12* mice. Data are shown as individual data points and mean ± SEM for n = 6 mice (v4 ABE7.10-NG-eVLP) or n = 4 mice (ABE7.10-NG-LV and untreated). p values were calculated using a two-sided t test. (G) Allele frequency distributions of genomic DNA harvested from treated *rd12* mice. Data are shown as mean ± SEM for n = 6 mice (v4 ABE7.10-NG-eVLP) or n = 4 mice (ABE7.10-NG-LV and untreated). 7.10-LV, ABE7.10-NG-LV; 7.10-eVLP, v4 ABE7.10-NG-eVLP. (H) Scotopic a- and b-wave amplitudes measured by ERG following overnight dark adaptation. Data are shown as individual data points and mean ± SEM for n = 8 mice (wild-type), n = 7 mice (v4 ABE7.10-NG-eVLP), n = 5 mice (ABE7.10-NG-LV), or n = 4 mice (untreated). p values were calculated using a two-sided t test. (I) Western blot of protein extracts from RPE tissues of wild-type, untreated, v4 ABE7.10-NG-eVLP-treated, and ABE7.10-NG-LV-treated mice. (J) Representative ERG waveforms from wild-type, untreated, ABE7.10-NG-LV-treated, and v4 ABE7.10-NG-eVLP-treated mice.

We designed and produced v4 BE-eVLPs encapsulating ABE8e-NG RNPs and an sgRNA (Figure 7A) that targets the *Rpe65*(R44X) mutation (hereafter referred to as ABE8e-NG-eVLPs). ABE8e-NG-eVLPs were pseudotyped with VSV-G to enable them to efficiently transduce retinal pigment epithelium (RPE) cells (Puppo et al., 2014; Suh et al., 2021). We injected ABE8e-NG-eVLPs subretinally into 4-week-old *rd12* mice. In a separate cohort, we also subretinally injected replication-incompetent lentivirus encoding the identical ABE8e-NG and sgRNA constructs (ABE8e-NG-LV). We previously reported that lentiviral delivery of ABEs can successfully restore visual function in *rd12* mice (Suh et al., 2021).

Five weeks post-injection, we harvested RPE tissue and performed high-throughput sequencing of RPE genomic DNA (Figure 7B). Encouragingly, sequencing analysis revealed that ABE8e-NG-eVLPs and ABE8e-NG-LV successfully mediated 21% and 11.5% correction, respectively, of the R44X mutation at position A_6_ of the protospacer (Figure 7C). Notably, ABE8e-NG-eVLPs achieved 1.8-fold higher editing at the target base compared to ABE8e-NG-LV, even though BE-eVLP delivery is transient. These results demonstrate that v4 BE-eVLPs enable highly efficient correction of a pathogenic mutation in mouse RPE.

While we observed highly efficient correction of the target mutation, we also observed that both ABE8e-NG-eVLP and ABE8e-NG-LV induced substantial levels of bystander editing (Figure 7C) due to the wide editing window of ABE8e-NG (Richter et al., 2020), such that the majority of edited alleles contained conversions at A_3_, A_6_, and/or A_8_ as opposed to A_6_ alone (Figure 7D). The bystander edits at positions A_3_ and A_8_ lead to *Rpe65* missense mutations C45R and L43P, respectively. We previously showed that the L43P mutation renders the *Rpe65* enzyme inactive (Suh et al., 2021). Indeed, after performing scotopic electroretinography (ERG) to assess retinal-cell response, we observed minimal rescue of visual function in both ABE8e-NG-eVLP-injected and ABE8e-NG-LV-injected eyes (Figure 7E). These results suggested that the wide base editing window of ABE8e-NG is not well suited to precisely correct the *Rpe65*(R44X) mutation.

To address this limitation, we designed and produced v4 BE-eVLPs that encapsulate ABE7.10-NG, which exhibits a narrower editing window compared to ABE8e-NG (Huang et al., 2019; Richter et al., 2020). Subretinal injection of ABE7.10-NG-eVLPs into adult *rd12* mice led to 12% correction of the R44X mutation in RPE genomic DNA with virtually no bystander editing (Figure 7F). Specifically, we observed that ABE7.10-NG-eVLP treatment resulted in 11% perfect R44X correction without bystander edits, a 9-fold improvement in perfect correction relative to ABE8e-NG-eVLP treatment (Figure 7G). Furthermore, treatment with ABE7.10-NG-eVLPs resulted in a 1.4-fold improvement in bystander-free correction relative to treatment with ABE7.10-NG-LV (a lentivirus encoding the identical ABE7.10-NG and sgRNA constructs), an additional demonstration that v4 BE-eVLP transient delivery can achieve comparable or higher editing efficiencies compared to lentiviral BE delivery (Figure 7G).

We confirmed via western blot that ABE7.10-NG-eVLP treatment restored the expression of Rpe65 protein. Notably, ABE7.10-NG-LV-treated eyes still expressed BE protein 5 weeks post-injection, while ABE7.10-NG-eVLP-treated eyes did not (Figure 7I), confirming the transient exposure of cells *in vivo* to BEs delivered using eVLPs. Importantly, ABE7.10-NG-eVLPs successfully rescued visual function to similar levels relative to ABE7.10-NG-LV as measured by ERG of the treated eyes (Figures 7H and 7J). We previously showed that this level of ERG rescue corresponds to other improvements in visual function, including restoration of the visual chromophore and recovery of visual cortical responses (Suh et al., 2021). These results demonstrate that eVLPs can mediate efficient correction of a pathogenic mutation in the mouse RPE with amelioration of the disease phenotype.

**Figure S7:**
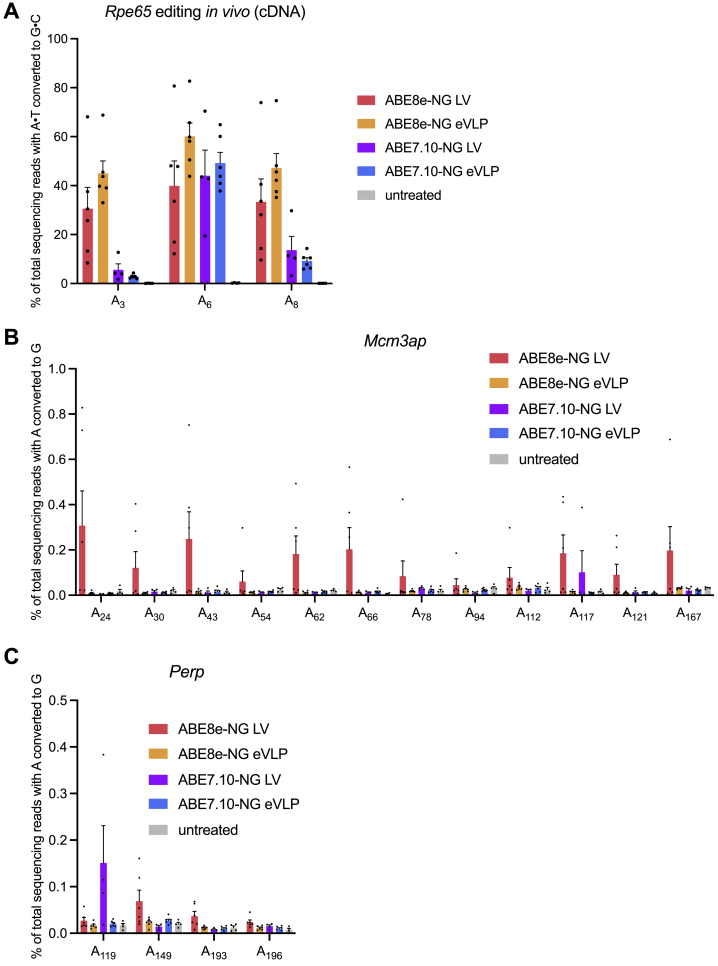
Sequencing analysis of RPE cDNA after v4 BE-eVLP or lentivirus treatment, related to Figure 7 (A) v4 BE-eVLP and lentivirus treatment led to 50%–60% of A⋅T-to-G⋅C conversion at the target adenine (A_6_) of the *Rpe65* transcript. Data are shown as individual data points and mean values ± SEM for n = 6 (ABE8e-NG-LV, ABE8e-NG-eVLP, and ABE7.10-NG-eVLP), or n = 4 (ABE7.10-NG-LV and untreated) mice. (B and C) Off-target A-to-G RNA editing by v4 BE-eVLPs and lentiviruses as measured by high-throughput sequencing of the (B) *Mcm3ap* and (C) *Perp* transcripts. Data are shown as individual data points and mean values ± SEM for n = 6 (ABE8e-NG-LV, ABE8e-NG-eVLP, and ABE7.10-NG-eVLP), or n = 4 (ABE7.10-NG-LV and untreated) mice.

To further analyze editing outcomes, we extracted RNA from treated eyes and performed targeted high-throughput sequencing of specific cDNAs. As expected, in the eVLP-treated eyes, we observed up to 64% of A⋅T-to-G⋅C conversion of the target adenine (A_6_) in the on-target *Rpe65* transcript (Figure S7A). The higher proportion of corrected *Rpe65* transcripts compared with *Rpe65* genomic loci potentially reflects nonsense-mediated decay of uncorrected mRNAs.

BEs are known to exhibit low-level transcriptome-wide Cas-independent off-target RNA editing (Anzalone et al., 2020). To investigate this possibility, we assessed off-target RNA editing by ABE-eVLPs and ABE-LVs by sequencing the *Mcm3ap* and *Perp* transcripts from treated eyes, two transcripts that were previously identified as potential candidates for off-target RNA editing based on their sequence similarity to the native TadA deaminase substrate (Jo et al., 2021). We observed RNA off-target editing by ABE8e-NG-LV in both transcripts and low but detectable RNA off-target editing by ABE7.10-NG-LV at one adenine in *Perp* (Figures S7B and S7C). In contrast, we did not detect any RNA off-target editing above background in these two transcripts by ABE8e-NG-eVLPs or ABE7.10-NG-eVLPs (Figures S7B and S7C). Collectively, these findings highlight the therapeutic utility of eVLPs as a DNA-free method for transiently delivering BE RNPs *in vivo* with high on-target editing and minimal off-target editing.

## Discussion

We have developed an efficient, engineered VLP platform that can safely deliver RNPs for therapeutically relevant *ex vivo* and *in vivo* applications. Through identifying and engineering solutions to three distinct bottlenecks to VLP-delivery efficiency, we improved protein loading within v4 eVLPs by an average of 16-fold and base editing efficiencies by an average of 8-fold compared with initial designs based on previously reported VLP scaffolds. Our findings suggest that v4 eVLPs are highly versatile and suitable for a wide range of both *ex vivo* and *in vivo* editing applications. We also anticipate that the eVLP architecture will serve as a modular platform for delivering other proteins or RNPs of interest, in addition to BEs and nucleases.

The results from our study highlight the potential therapeutic benefit of using rational engineering to further advance delivery platforms for gene editing agents. While VLPs have been used previously to deliver Cas9 nuclease RNPs (Campbell et al., 2019; Choi et al., 2016; Gee et al., 2020; Hamilton et al., 2021; Indikova and Indik, 2020; Lyu et al., 2019; Mangeot et al., 2019), and a recent study used VLPs to deliver BE RNPs to HEK293T cells with lower efficiencies than the eVLPs described here (Lyu et al., 2021), no previous study to our knowledge has reported therapeutic levels of postnatal *in vivo* gene editing of any type using RNP-delivering VLPs. The eVLP platform developed in this work uses a rationally engineered architecture that we customized to package increased amounts of cargo and improve particle titers. These eVLPs can mediate therapeutic levels of *in vivo* base editing across multiple organs and routes of administration in mice, achieving the highest levels of postnatal *in vivo* gene editing using RNPs reported to date.

A single intravenous injection of eVLPs mediated base editing of *Pcsk9* in the mouse liver at efficiencies >60%, comparable to those achieved at the same target by current state-of-the-art BE delivery methods, including AAV-mediated delivery of BE-encoding DNA (Rothgangl et al., 2021) and LNP-mediated delivery of BE-encoding mRNA (Musunuru et al., 2021; Rothgangl et al., 2021). However, eVLPs offer key advantages over both AAV-mediated DNA delivery and LNP-mediated mRNA delivery strategies. AAV-mediated delivery can lead to detectable levels of viral genome integration into the genomes of transduced cells, which can lead to oncogenesis (Chandler et al., 2017; Koblan et al., 2021), while eVLPs lack DNA and therefore should avoid the possibility of insertional mutagenesis. Additionally, AAV-mediated delivery leads to prolonged cargo expression, which can increase the frequency of off-target editing when delivering gene editing agents. In contrast, transient eVLP-mediated delivery of BE RNPs greatly reduces the opportunity for off-target editing, as we showed both *in vitro* and *in vivo* (Figures 3G, 3H, and 6D). While LNP-mediated delivery of BE-encoding mRNA is also transient, delivering BE RNPs offers even shorter exposures to editing agents and lower off-target editing opportunities due to the shorter lifetime of RNPs in cells compared with mRNA, each copy of which generates cellular RNPs throughout the lifetime of the mRNA (Newby et al., 2021).

While LNPs can efficiently package mRNAs, packaging gene editing agent RNPs within LNPs is substantially more challenging (Wei et al., 2020). Because eVLPs can achieve comparable levels of editing in the liver as these other strategies, but possess the important advantages mentioned above, they are a particularly attractive option for further development as a therapeutic modality for *in vivo* editing approaches to treat genetic liver diseases. The v4 BE-eVLP architecture was critical for achieving robust editing in the mouse liver and improved *in vivo* editing efficiency by 26-fold compared with a previously reported (v1) VLP design (Figure 6B), underscoring the importance of engineering VLP architectures for *in vivo* editing. The observed degree of base editing at this *Pcsk9* splice donor with v4 BE-eVLPs (>60%) is thought to be sufficient for the reduction of serum LDL and treatment of hypercholesterolemia (Musunuru et al., 2021).

A single subretinal injection of v4 BE-eVLPs in a mouse model of LCA efficiently corrected the disease-causing point mutation and restored visual function. In this model, once again, eVLPs achieved editing efficiencies and levels of rescue that are comparable with or higher than those previously achieved using viral delivery methods, including lentiviral BE delivery (Suh et al., 2021) and AAV-mediated BE delivery (Jo et al., 2021). The accessibility of the eyes and their immune-privileged status (Taylor, 2009) may more readily enable the translation of new delivery modalities into preclinical and clinical studies. Our data provide evidence of the therapeutic potential of BE-eVLPs as a means to correct pathogenic point mutations that cause ocular disorders.

The developments reported here combine the one-time treatment potential of gene editing agents and the transient nature of RNPs to minimize the opportunity for unwanted off-target editing or DNA integration with the efficient, tissue-targeted nature of viral transduction. Our findings thus suggest eVLPs as an attractive alternative to other delivery strategies for both *in vivo* or *ex vivo* delivery of BEs, nucleases, and other proteins of therapeutic interest.

### Limitations of the study

The eVLPs are produced following co-transfection of producer cells (Gesicle HEK293T) with cargo (BE), gag–pro-pol, envelope, and sgRNA plasmids. During production, eVLPs bud out from the cytoplasm of producer cells and may, therefore, nonspecifically package cellular proteins and RNAs that are highly expressed in the producer cells. The nonspecific co-packaging of cellular proteins has also been observed previously for some clinical-grade lentiviral vectors produced using similar methods (Johnson et al., 2018; Wheeler et al., 2007). A previous study found that some cellular membrane-associated proteins, tRNAs, rRNAs, and mRNAs are co-packaged into MLV-based Cas9-VLPs produced from the same gesicle HEK293T producer cell line (Mangeot et al., 2011, 2019). It is likely that similar cellular contents may be co-packaged within eVLPs. Although BE-eVLP treatment was nontoxic both *in vitro* and *in vivo*, additional studies to fully characterize the protein and RNA contents of eVLPs and their effects on transduced cells will help improve our understanding of this delivery system.

While we established that eVLPs enable efficient *in vivo* base editing in multiple organs, future efforts to improve editing efficiencies in other tissues could further expand the therapeutic potential of eVLPs. In addition, we showed that eVLPs offer transient delivery of BEs, though the precise half-life of the cargo after *in vivo* delivery remains unknown. Future pharmacokinetic studies in mice and nonhuman primates will help determine the residence time of eVLPs, their cargoes, and dosing requirements. Additional characterization of eVLPs, including contents that may be packaged from the producer cells, would be helpful to evaluate and mitigate their potential immunogenicity.

## STAR★Methods

### Key resources table

**Table:** 

REAGENT or RESOURCE	SOURCE	IDENTIFIER
**Antibodies**		

Mouse anti-Cas9 monoclonal antibody	Thermo Fisher Scientific	Cat#MA5-23519; RRID:AB_2610639
Mouse anti-MLV p30 monoclonal antibody	Abcam	Cat#ab130757
Mouse anti-VSVG monoclonal antibody	Sigma-Aldrich	Cat#V5507
IRDye 680RD goat anti-mouse antibody	LI-COR	Cat#926-68070; RRID:AB_10956588
Mouse anti-Cas9 monoclonal antibody	Cell Signaling Technology	Cat#14697; RRID:AB_2750916
Rabbit anti-tubulin monoclonal antibody	Abcam	Cat#ab52866; RRID:AB_869989
Goat anti-mouse AF647-conjugated antibody	Abcam	Cat#ab150115; RRID:AB_2687948
Goat anti-rabbit AF488-conjugated antibody	Abcam	Cat#ab150077; RRID:AB_2630356
Mouse anti-Rpe65 monoclonal antibody	(Golczak et al., 2010)	
Goat anti-mouse IgG-HRP antibody	Cell Signaling Technology	Cat#7076S; RRID:AB_330924
Rabbit anti-β-actin polyclonal antibody	Cell Signaling Technology	Cat#4970S; RRID:AB_2223172
Goat anti-rabbit IgG-HRP antibody	Cell Signaling Technology	Cat#7074S; RRID:AB_2099233

**Bacterial and virus strains**		

One Shot Mach1 T1 Phage-Resistant Chemically Competent *E. coli*	Thermo Fisher Scientific	Cat#C862003
NEB Stable Competent *E. coli*	New England BioLabs	Cat#C3040H

**Chemicals, peptides, and recombinant Proteins**		

USER enzyme	New England BioLabs	Cat#M5505S
DpnI	New England BioLabs	Cat#R0176S
KLD Enzyme Mix	New England BioLabs	Cat#M0554S
Lipofectamine 2000	Thermo Fisher Scientific	Cat#11668019
jetPRIME Transfection Reagent	Polyplus	Cat#114-75
FuGENE HD Transfection Reagent	Promega	Cat#E2312
PEG-it Virus Precipitation Solution	System Biosciences	Cat#LV825A-1
Recombinant Cas9 (*S. pyogenes*) nuclease	New England BioLabs	Cat#M0386
SYBR green dye	Lonza	Cat#50512
Proteinase K	Thermo Fisher Scientific	Cat#EO0492
Proteinase K	New England BioLabs	Cat#P8107S
Human AB Serum	Valley Biomedical	Cat#HP1022HI
*N*-Acetyl-L-cysteine	Sigma-Aldrich	Cat#A7250-100G
Recombinant Human IL-2	Peprotech	Cat#200-02
Recombinant Human IL-7	Peprotech	Cat#200-07
Recombinant Human IL-15	Peprotech	Cat#200-15
RetroNectin	Clontech/Takara	Cat#T100A/B
Dynabeads Human T-Expander CD3/CD28 beads	Thermo Fisher Scientific	Cat#1161D
QuickExtract DNA Extraction Solution	Lucigen	Cat#QE09050
Salt Active Nuclease	ArcticZymes	Cat#70910-202
BSA	New England BioLabs	Cat#B9000S
0.9% NaCl	Fresenius Kabi	Cat#918610

**Critical commercial assays**		

Phusion U Multiplex PCR Master Mix	Thermo Fisher Scientific	Cat#F562L
Phusion High-Fidelity DNA Polymerase	New England BioLabs	Cat#M0530S
QIAquick PCR Purification Kit	QIAGEN	Cat#28104
QIAquick Gel Extraction Kit	QIAGEN	Cat#28704
QIAGEN Plasmid *Plus* Midi Kit	QIAGEN	Cat#12943
QIAGEN Plasmid *Plus* Maxi Kit	QIAGEN	Cat#12963
FastScan Cas9 (*S. pyogenes*) ELISA Kit	Cell Signaling Technology	Cat#29666C
MuLV Core Antigen ELISA Kit	Cell Biolabs	Cat#VPK-156
QIAmp Viral RNA Mini Kit	QIAGEN	Cat#52904
SuperScript III First-Strand Synthesis SuperMix	Thermo Fisher Scientific	Cat#18080400
EasySep Human T Cell Isolation Kit	STEMCELL Technologies	Cat#17951
AAVpro Titration Kit version 2	Clontech/Takara	Cat#6233
Agencourt DNAdvance Kit	Beckman	Cat#V10309
CellTiter-Glo Luminescent Cell Viability Kit	Promega	Cat#G7570
Mouse Proprotein Convertase 9/PCSK9 Quantikine ELISA Kit	R&D Systems	Cat#MPC900
QuickTiter Lentivirus Titer Kit	Cell Biolabs	Cat#VPK-107-5
AllPrep DNA/RNA Mini Kit	QIAGEN	Cat#80284
MiSeq Reagent Kit v2 (300-cycles)	Illumina	Cat#MS-102-2002
MiSeq Reagent Micro Kit v2 (300-cycles)	Illumina	Cat#MS-103-1002

**Deposited data**		

Targeted amplicon sequencing data	This study	PRJNA768458

**Experimental models: Cell lines**		

Human: HEK293T	ATCC	Cat#CRL-3216
Human: Gesicle Producer 293T	Takara	Cat#632617
Mouse: NIH/3T3	ATCC	Cat#CRL-1658
Mouse: Neuro-2a	ATCC	Cat#CCL-131

**Experimental models: Organisms**		

Timed pregnant C57BL/6J mice	Charles River Laboratories	Cat#027
C57BL/6J mice	Jackson Laboratory	Cat#000664
*rd12* mice	Jackson Laboratory	Cat#005379

**Recombinant DNA**		

pCMV-VSV-G	Addgene	8454
psPAX2	Addgene	12260
pBS-CMV-gagpol	Addgene	35614
BIC-Gag-Cas9	Addgene	119942
lentiCRISPRv2	Addgene	135955
v4 BE-eVLP	Addgene (this study)	TBA

**Software and algorithms**		

CRISPResso2	(Clement et al., 2019)	https://github.com/pinellolab/CRISPResso2
Prism	GraphPad	https://www.graphpad.com

### Resource availability

#### Lead contact

Please direct requests for resources and reagents to Lead Contact: David R. Liu (D.R.L. drliu@fas.harvard.edu)

#### Materials availability

Plasmids generated in this study are available from Addgene (additional details provided in the Key Resources Table).

### Experimental model and subject details

#### Cell culture conditions

HEK293T cells (ATCC; CRL-3216), Gesicle Producer 293T cells (Takara; 632617), 3T3 cells (ATCC; CRL-1658), and Neuro-2a cells (ATCC; CCL-131) were maintained in DMEM + GlutaMAX (Life Technologies) supplemented with 10% (v/v) fetal bovine serum. Primary human and mouse fibroblasts were maintained in MEM alpha media (Thermo Fisher; 12571063) containing 20% (v/v) FBS, 2 mM GlutaMAX (Thermo Fisher; 35050061), 1 % penicillin and streptomycin (Thermo Fisher; 15070063), 1X Nonessential amino acids (Thermo Fisher; 11140050), 1X Antioxidant Supplement (Sigma Aldrich; A1345), 10 ng/mL Epidermal Growth Factor from murine submaxillary gland (Sigma Aldrich; E4127) and 0.5 ng/mL Fibroblast Growth Factor (Sigma Aldrich; F3133). Cells were cultured at 37 °C with 5% carbon dioxide and were confirmed to be negative for mycoplasma by testing with MycoAlert (Lonza Biologics).

#### Isolation of primary human T cells

Primary human T cells were isolated as described previously (Chen et al., 2021). Buffy coats were obtained from Memorial Blood Centers (St. Paul, MN) and peripheral blood mononuclear cells were isolated using SepMate tubes (STEMCELL Technologies; 85450). The EasySep Human T-cell Isolation Kit was used to enrich for T-cells that were then frozen for long-term storage.

### Method details

#### Cloning

All plasmids used in this study were cloned using either USER cloning or KLD cloning as described previously (Doman et al., 2020). DNA was PCR-amplified using PhusionU Green Multiplex PCR Master Mix (Thermo Fisher Scientific). Mach1 (Thermo Fisher Scientific) chemically competent *E. coli* were used for plasmid propagation.

#### BE-eVLP production and purification

BE-eVLPs were produced by transient transfection of Gesicle Producer 293T cells. Gesicle cells were seeded in T-75 flasks (Corning) at a density of 5×10^6^ cells per flask. After 20–24 h, cells were transfected using the jetPRIME transfection reagent (Polyplus) according to the manufacturer’s protocols. For producing v1–v3 BE-eVLPs, a mixture of plasmids expressing VSV-G (400 ng), MLVgag–pro–pol (2,800 ng), MLVgag–ABE8e (1,700 ng), and an sgRNA (4,400 ng) were co-transfected per T-75 flask. For MLVgag–ABE8e:MLVgag–pro–pol stoichiometry optimization, the total amount of plasmid DNA for these two components was fixed at 4,500 ng and the relative amounts of each were varied. For producing v4 BE-eVLPs, a mixture of plasmids expressing VSV-G (400 ng), MMLVgag–pro–pol (3,375 ng), MMLVgag–3xNES–ABE8e (1,125 ng), and an sgRNA (4,400 ng) were co-transfected per T-75 flask. BE-eVLP construct protein sequences are provided in Table S1.

40–48 h post-transfection, producer cell supernatant was harvested and centrifuged for 5 min at 500 *g* to remove cell debris. The clarified eVLP-containing supernatant was filtered through a 0.45-μm PVDF filter. For BE-eVLPs that were used in cell culture, unless otherwise stated, the filtered supernatant was concentrated 100-fold using PEG-it Virus Precipitation Solution (System Biosciences; LV825A-1) according to the manufacturer’s protocols. For BE-eVLPs that were injected into mice, the filtered supernatant was concentrated 1000–3000-fold by ultracentrifugation using a cushion of 20% (w/v) sucrose in PBS. Ultracentrifugation was performed at 26,000 rpm for 2 h (4°C) using either an SW28 rotor in an Optima XPN Ultracentrifuge (Beckman Coulter) or an AH-629 rotor in a Sorvall WX+ Ultracentrifuge (Thermo Fisher Scientific). Following ultracentrifugation, BE-eVLP pellets were resuspended in cold PBS (pH 7.4) and centrifuged at 1,000 *g* for 5 min to remove debris. BE-eVLPs were frozen at a rate of -1°C/min and stored at -80°C. BE-VLPs were thawed on ice immediately prior to use.

#### BE-eVLP transduction in cell culture and genomic DNA isolation

Cells were plated for transduction in 48-well plates (Corning) at a density of 30,000–40,000 cells per well. After 20–24 h, BE-eVLPs were added directly to the culture media in each well. 48–72 h post-transduction, cellular genomic DNA was isolated as previously reported (Doman et al., 2020). Briefly, cells were washed once with PBS and lysed in 150 μL of lysis buffer (10 mM Tris-HCl pH 8.0, 0.05% SDS, 25 μg mL^-1^ Proteinase K (Thermo Fisher Scientific)) at 37 °C for 1 h followed by heat inactivation at 80°C for 30 min.

#### High-throughput sequencing of genomic DNA

Genomic DNA was isolated as described above. Following genomic DNA isolation, 1 μL of the isolated DNA (1–10 ng) was used as input for the first of two PCR reactions. Genomic loci were amplified in PCR1 using PhusionU polymerase (Thermo Fisher Scientific). PCR1 primers for genomic loci are listed in Table S2 under the HTS_fwd and HTS_rev columns. PCR1 was performed as follows: 95 °C for 3 min; 30–35 cycles of 95 °C for 15 s, 61 °C for 20 s, and 72 °C for 30s; 72°C for 1 min. PCR1 products were confirmed on a 1% agarose gel. 1 μL of PCR1 was used as an input for PCR2 to install Illumina barcodes. PCR2 was conducted for nine cycles of amplification using a Phusion HS II kit (Life Technologies). Following PCR2, samples were pooled and gel purified in a 1% agarose gel using a Qiaquick Gel Extraction Kit (Qiagen). Library concentration was quantified using the Qubit High-Sensitivity Assay Kit (Thermo Fisher Scientific). Samples were sequenced on an Illumina MiSeq instrument (paired-end read, read 1: 200–280 cycles, read 2: 0 cycles) using an Illumina MiSeq 300 v2 Kit (Illumina).

#### High-throughput sequencing data analysis

Sequencing reads were demultiplexed using the MiSeq Reporter software (Illumina) and were analyzed using CRISPResso2 (Clement et al., 2019) as previously described (Doman et al., 2020). Batch analysis mode (one batch for each unique amplicon and sgRNA combination analyzed) was used in all cases. Reads were filtered by minimum average quality score (Q > 30) prior to analysis. The following quantification window parameters were used: -w 20 -wc -10. Base editing efficiencies are reported as the percentage of sequencing reads containing a given base conversion at a specific position. Prism 9 (GraphPad) was used to generate dot plots and bar plots.

#### Immunoblot analysis of BE-eVLP protein content

BE-eVLPs were lysed in Laemmli sample buffer (50 mM Tris-HCl pH 7.0, 2% sodium dodecyl sulfate (SDS), 10% (v/v) glycerol, 2 mM dithiothreitol (DTT)) by heating at 95°C for 15 min. Lysed BE-eVLPs were spotted onto a dry nitrocellulose membrane (Thermo Fisher Scientific) and dried for 30 min. The membrane was blocked for 1 h at room temperature with rocking in blocking buffer: 1% bovine serum albumin (BSA) in TBST (150 mM NaCl, 0.5% Tween-20, and 50 mM Tris-HCl). After blocking, the membrane was incubated overnight at 4°C with rocking with one of the following primary antibodies diluted in blocking buffer: mouse anti-Cas9 (Thermo Fisher; MA5-23519, 1:1000 dilution), mouse anti-MLV p30 (Abcam; ab130757, 1:1500 dilution), or mouse anti-VSV-G (Sigma Aldrich; V5507, 1:50000 dilution). The membrane was washed three times with 1xTBST (Tris-buffered saline + 0.5% Tween-20) for 10 min each time at room temperature, then incubated with goat anti-mouse antibody (LI-COR IRDye 680RD; 926-68070, 1:10000 dilution in blocking buffer) for 1 h at room temperature with rocking. The membrane was washed as before and imaged using an Odyssey Imaging System (LI-COR).

#### Western blot analysis of BE-eVLP protein content

BE-eVLPs were lysed as described above. Protein extracts were separated by electrophoresis at 150 V for 45 min on a NuPAGE 3–8% Tris-Acetate gel (Thermo Fisher Scientific) in NuPAGE Tris-Acetate SDS running buffer (Thermo Fisher Scientific). Transfer to a PVDF membrane was performed using an iBlot 2 Gel Transfer Device (Thermo Fisher Scientific) at 20 V for 7 min. The membrane was blocked for 1 h at room temperature with rocking in blocking buffer: 1% bovine serum albumin (BSA) in TBST (150 mM NaCl, 0.5% Tween-20, and 50 mM Tris-HCl). After blocking, the membrane was incubated overnight at 4°C with rocking with mouse anti-Cas9 (Cell Signaling Technology; 14697, 1:1000 dilution). The membrane was washed three times with 1xTBST for 10 min each time at room temperature, then incubated with goat anti-mouse antibody (LI-COR IRDye 680RD; 926-68070, 1:10000 dilution in blocking buffer) for 1 h at room temperature with rocking. The membrane was washed as before and imaged using an Odyssey Imaging System (LI-COR). The relative amounts of cleaved ABE and full-length gag–ABE were quantified by densitometry using ImageJ, and the fraction of cleaved ABE relative to total (cleaved + full-length) ABE was calculated.

#### Immunofluorescence microscopy of producer cells

Gesicle Producer 293T cells were seeded at a density of 15,000 cells per well in PhenoPlate™ 96-well microplates coated with poly-D-lysine (PerkinElmer). After 24 h, cells were co-transfected with 1 ng of v2.4 or v3.4 BE-eVLP plasmids, 40 ng of mouse *Dnmt1*-targeting sgRNA plasmid, and 40 ng of pUC19 plasmid using the jetPRIME transfection reagent (Polyplus) according to the manufacturer’s protocols. After 40 h, 32% aqueous paraformaldehyde (Electron Microscopy Sciences) was added dropwise directly into the cellular media to a final concentration of 4% paraformaldehyde. Cells were subsequently fixed for 20 min at room temperature. After fixation, cells were washed three times with PBS and then permeabilized with 1xPBST (PBS + 0.1% Triton X-100) for 30 min at room temperature. Cells were then blocked in blocking buffer (3% w/v BSA in 1xPBST) for 30 min at room temperature. After blocking, cells were incubated overnight at 4°C with mouse anti-Cas9 (Cell Signaling Technology; 14697, 1:250 dilution) and rabbit anti-tubulin (abcam; 52866, 1:400 dilution) diluted in blocking buffer. Cells were washed four times with 1xPBST, then incubated for 1 h at room temperature with goat anti-mouse AlexaFluor® 647-conjugated antibody (abcam; 150115, 1:500 dilution), goat anti-rabbit AlexaFluor® 488-conjugated antibody (abcam; 150077, 1:500 dilution), and 1 μM DAPI diluted in blocking buffer. Cells were washed three times with 1xPBST and two times with PBS before imaging using an Opera Phenix High-Content Screening System (PerkinElmer). Images were acquired using a 20x water immersion objective in a confocal mode. Automated image analysis was performed using the Harmony software (PerkinElmer). The normalized cytoplasmic intensity was determined by calculating the ratio of the mean cytoplasmic intensity of Cas9 signal per cell to the mean cytoplasmic intensity of tubulin signal per cell.

#### Negative-stain transmission electron microscopy

Negative-stain TEM was performed at the Koch Nanotechnology Materials Core Facility of MIT. v4 BE-eVLPs were centrifuged for 5 min at 15,000 *g* to remove debris. From the clarified supernatant, 10 μL of sample and buffer containing solution was added to 200 mesh copper grid coated with a continuous carbon film. The sample was allowed to adsorb for 60 seconds after which excess solution was removed with kimwipes. 10 μL of negative staining solution containing 1% aqueous phosphotungstic acid was added to the TEM grid and the stain was immediately blotted off with kimwipes. The grid was then air-dried at room temperature in the chemical hood. The grid was then mounted on a JEOL single tilt holder equipped within the TEM column. The specimen was cooled down by liquid-nitrogen and then observed using JEOL 2100 FEG microscope at 200kV with a magnification of 10,000–60,000. Images were taken using Gatan 2kx2k UltraScan CCD camera.

#### BE-eVLP protein content quantification

For protein quantification, BE-eVLPs were lysed in Laemmli sample buffer as described above. The concentration of BE protein in purified BE-eVLPs was quantified using the FastScan™ Cas9 (*S. pyogenes*) ELISA kit (Cell Signaling Technology; 29666C) according to the manufacturer’s protocols. Recombinant Cas9 (*S. pyogenes*) nuclease protein (New England Biolabs; M0386) was used to generate the standard curve for quantification. The concentration of MLV p30 protein in purified BE-eVLPs was quantified using the MuLV Core Antigen ELISA kit (Cell Biolabs; VPK-156) according to the manufacturer’s protocols. The concentration of VLP-associated p30 protein was calculated with the assumption that 20% of the observed p30 in solution was associated with VLPs, as was previously reported for MLV particles (Renner et al., 2020). The number of BE protein molecules per eVLP was calculated by assuming a copy number of 1800 molecules of p30 per eVLP, as was previously reported for MLV particles (Renner et al., 2020). The same analysis was used to determine eVLP titers for all therapeutic application experiments.

#### BE-eVLP sgRNA extraction and quantification

RNA was extracted from BE-eVLPs using the QIAmp Viral RNA Mini Kit (Qiagen; 52904) according to the manufacturer’s protocols. Extracted RNA was reverse transcribed using SuperScript™ III First-Strand Synthesis SuperMix (Thermo Fisher Scientific; 18080400) and an sgRNA-specific DNA primer (Table S3) according to the manufacturer’s protocols. qPCR was performed using a CFX96 Touch Real-Time PCR Detection System (Bio-Rad) with SYBR green dye (Lonza; 50512). The amount of cDNA input was normalized to MLV p30 content, and the sgRNA abundance per eVLP was calculated as log_2_ (ΔC_q_) relative to v1 BE-VLPs.

#### Cell viability assays

Cell viability was quantified using a Promega CellTiter-Glo luminescent cell viability kit (Promega; G7570). 4x10^4^ cells (for HEK293T and NIH 3T3) and 2.5x10^4^ cells (for RDEB patient fibroblasts) were seeded in 250 μL of media per well. The cells were allowed to adhere for 16-18 h before treatment with BE-eVLPs. After 48 h of transduction, 100 μL of CellTiter-Glo reagent was added to each well in the dark. Cells were incubated for 10 min at room temperature and the 80 μL of solution was transferred into black 96-well flat bottom plates (Greiner Bio-one; 655096), and the luminescence was measured on a M1000 Pro microplate reader (Tecan) with a 1-second integration time. Cells treated with Opti-MEM were defined as 100% viable. The percentage of viable cells in BE-eVLP treated wells was calculated by normalizing the luminescence reading from each treatment well to the luminescence of PBS treated cells.

#### Plasmid transfections

Plasmid transfections were performed as described previously (Doman et al., 2020). Plasmids were prepared for transfection using a PlasmidPlus Midi Kit (Qiagen) with endotoxin removal. HEK293T cells were plated for transfection in 48-well plates (Corning) at a density of 40,000 cells per well. After 20–24 h, cells were transfected with 1 μg total DNA using 1.5 μL of Lipofectamine 2000 (Thermo Fisher Scientific) per well according to the manufacturer’s protocols. Unless otherwise specified, 750 ng of base editor plasmid and 250 ng of guide RNA plasmid were co-transfected per well. Genomic DNA was isolated from transfected cells at 72 h post-transfection as described above.

#### Assessment of off-target DNA base editing in HEK293T cells

HEK293T cells were transduced with v4 BE-eVLPs or transfected with BE-encoding plasmid as described above. To assess Cas-dependent off-target editing, cells were transfected or transduced with 1 μL of v4 BE-eVLPs on the same day and genomic DNA was isolated 72 h post treatment in both cases. On-target and off-target loci were amplified and sequenced as described above. Orthogonal R-loop assays were performed as described previously (Doman et al., 2020) to assess Cas-independent off-target editing. To allow time for expression of SaCas9 and formation of the off-target R-loops following plasmid transfection, cells were transduced with 1 μL of PEG-concentrated v4 BE-eVLPs at 24 h post-transfection with dSaCas9- and orthogonal sgRNA-encoding plasmids. Genomic DNA was isolated 72 h post-transfection (48 h post-transduction) and sequenced as described above. See also Figure S4A for an experimental schematic.

#### Quantification of BE-encoding DNA

For quantifying the amount of BE-encoding DNA in BE-eVLP preparations, v4 BE-eVLPs were lysed as described above, and the lysate was used as input into a qPCR reaction with BE-specific primers (Table S3). For quantifying the amount of BE-encoding DNA in eVLP-transduced vs. plasmid-transfected HEK293T cells, DNA was isolated from cell lysate as described above and used as input into a qPCR reaction with BE-specific primers (Table S3). In both cases, a standard curve was generated with BE-encoding plasmid standards of known concentration and was used to infer the amount of BE-encoding DNA present in the original samples.

#### Transduction of T cells and genomic DNA preparation

Thawed cells (day 0) were rested for 24 h in basal T-cell media comprised of X-VIVO™ 15 Serum-free Hematopoietic Cell Medium (Lonza; BE02-0606F) with 10% AB human serum (Valley Biomedical; HP1022), 2 mg/mL N-acetyl-cysteine (Sigma Aldrich; A7250), 300 IU/mL recombinant human IL-2 (Peprotech ; 200-02) and 5 ng/mL recombinant human IL-7 (Peprotech ; 200-07) and 5 ng/mL IL-15 (Peprotech; 500-P15). On day 1, 50,000 cells in 50 μL of T-cell media were plated in 96-well-plates coated with 10 μg/cm^2^ RectroNectin® (Clontech/Takara; catalog number T100A/B). 5 μL (3.0x10^10^ eVLPs) of ultracentrifuge-purified v4 BE-eVLPs were used to transduce the cells on day 1 and on day 2 the cells were stimulated with Dynabeads™ Human T-Expander CD3/CD28 beads (Thermo Fisher; 11161D). Beads were added at a bead to cell ratio of 3:1 in a volume of 50 μL. On day 3, the cells were transduced for a second time with 5 μL (3.0x10^10^ eVLPs) of v4 BE-eVLPs in a total media volume of 200 μL. Twenty-four hours later (day 4) the cells were resuspended in 1 mL of fresh T-cell media and re-plated in wells of a 48 well plate. On day 6 the cells were harvested and genomic DNA was isolated using the QuickExtract™ DNA Extraction Solution (Lucigen; QE09050).

#### Lentiviral vector cloning and production

Lentiviral vectors were constructed via USER cloning into the lentiCRISPRv2 backbone (Addgene #135955). Lentiviral transfer vectors were propagated in NEB Stable Competent *E. coli* (New England Biolabs). HEK293T/17 (ATCC CRL-11268) cells were maintained in antibiotic-free DMEM supplemented with 10% fetal bovine serum (v/v). On day 1, 5×10^6^ cells were plated in 10 mL of media in T75 flasks. The following day, cells were transfected with 6 μg of VSV-G envelope plasmid, 9 μg of psPAX2 (plasmid encoding viral packaging proteins) and 9 μg of transfer vector plasmid (plasmid encoding the gene of interest) diluted in 1,500 μL Opti-MEM with 70 μL of FuGENE. Two days after transfection, media was centrifuged at 500 *g* for 5 min to remove cell debris following filtration using 0.45-μm PVDF vacuum filter. The lentiviruses were further concentrated by ultracentrifugation with a 20% (w/v) sucrose cushion as described above for eVLP production.

#### AAV production

AAV production was performed as previously described (Deverman et al., 2016; Levy et al., 2020) with some alterations. HEK293T/17 cells were maintained in DMEM with 10% fetal bovine serum without antibiotics in 150-mm dishes (Thermo Fisher Scientific; 157150) and passaged every 2–3 days. Cells for production were split 1:3 one day before polyethylenimine transfection. Then, 5.7 μg AAV genome, 11.4 μg pHelper (Clontech) and 22.8 μg AAV8 rep-cap plasmid were transfected per plate. The day after transfection, media was exchanged for DMEM with 5% fetal bovine serum. Three days after transfection, cells were scraped with a rubber cell scraper (Corning), pelleted by centrifugation for 10 min at 2,000 *g*, resuspended in 500 μl hypertonic lysis buffer per plate (40 mM Tris base, 500 mM NaCl, 2 mM MgCl_2_ and 100 U mL^−1^ salt active nuclease (ArcticZymes; 70910-202)) and incubated at 37 °C for 1 h to lyse the cells. The media was decanted, combined with a 5X solution of 40% poly(ethylene glycol) (PEG) in 2.5 M NaCl (final concentration: 8% PEG/500 mM NaCl), incubated on ice for 2 h to facilitate PEG precipitation, and centrifuged at 3,200 *g* for 30 min. The supernatant was discarded and the pellet was resuspended in 500 μL lysis buffer per plate and added to the cell lysate. Crude lysates were either incubated at 4 °C overnight or directly used for ultracentrifugation.

Cell lysates were clarified by centrifugation at 2,000 *g* for 10 min and added to Beckman Quick-Seal tubes via 16-gauge 5” disposable needles (Air-Tite N165). A discontinuous iodixanol gradient was formed by sequentially floating layers: 9 mL 15% iodixanol in 500 mM NaCl and 1× PBS-MK (1× PBS plus 1 mM MgCl_2_ and 2.5 mM KCl), 6 mL 25% iodixanol in 1× PBS-MK, and 5 mL each of 40 and 60% iodixanol in 1× PBS-MK. Phenol red at a final concentration of 1 μg mL^−1^ was added to the 15, 25 and 60% layers to facilitate identification. Ultracentrifugation was performed using a Ti 70 rotor in a Optima XPN-100 Ultracentrifuge (Beckman Coulter) at 58,600 rpm for 2 h 15 min at 18 °C. Following ultracentrifugation, 3 mL of solution was withdrawn from the 40–60% iodixanol interface via an 18-gauge needle, dialyzed with PBS containing 0.001% F-68 using 100-kD MWCO columns (EMD Millipore). The concentrated viral solution was sterile filtered using a 0.22-μm filter. The final AAV preparation was quantified via qPCR (AAVpro Titration Kit version 2; Clontech), and stored at 4 °C until use.

#### Animals

All mice experiments were approved by the Broad Institute, the University of California, Irvine, and the University of Pennsylvania institutional animal care and use committees. Timed pregnant C57BL/6J mice for P0 studies were purchased from Charles River Laboratories (027). Wild-type adult C57BL/6J mice (000664) and pigmented *rd12* mice (005379) were purchased from the Jackson Laboratory. All mice were housed in a room maintained on a 12 h light and dark cycle with *ad libitum* access to standard rodent diet and water. Animals were randomly assigned to various experimental groups.

#### P0 ventricle injections

P0 ventricle injections were performed as described previously (Levy et al., 2020). Drummond PCR pipettes (5-000-1001-X10) were pulled at the ramp test value on a Sutter P1000 micropipette puller and passed through a Kimwipe three times, resulting in a tip size of ∼100 μm. A small amount of Fast Green was added to the BE-eVLP injection solution to assess ventricle targeting. The injection solution was loaded via front filling using the included Drummond plungers. P0 pups were anaesthetized by placement on ice for 2–3 min until they were immobile and unresponsive to a toe pinch. Then, 2 μL of injection mix (containing 2.6x10^10^ eVLPs encapsulating a total of 3.2 pmol of BE protein) was injected freehand into each ventricle. Ventricle targeting was assessed by the spread of Fast Green throughout the ventricles via transillumination of the head.

#### Nuclear isolation and sorting

Nuclei were isolated from the cortex and the mid-brain as previously described (Levy et al., 2020). Briefly, dissected cortex and mid-brain were homogenized using a glass Dounce homogenizer (Sigma-Aldrich; D8938) with 20 strokes using pestle A followed by 20 strokes from pestle B in 2 mL of ice-cold EZ-PREP buffer (Sigma-Aldrich; NUC-101). Samples were then decanted into a new tube containing an additional 2 mL of EZ-PREP buffer on ice. After 5 min, homogenized tissues were centrifuged for 5 min at 500 *g* at 4^°^C. The nuclei pellet was resuspended in 4 mL of ice-cold Nuclei Suspension Buffer (NSB) consisting of 100 μg/mL BSA (NEB; B9000S) and 3.33 μM Vybrant DyeCycle Ruby (Thermo Fisher; V10309) in PBS followed by centrifugation at 500 *g* for 5 min at 4^°^C. After centrifugation, the supernatant was removed, and nuclei were resuspended in 1-2 mL of NSB, passed through 35-μm cell strainer, followed by flow sorting using the Sony MA900 Cell Sorter (Sony Biotechnology) at the Broad Institute flow cytometry core. See Figure S5A for example FACS gating. Nuclei were sorted into DNAdvance lysis buffer, and the genomic DNA was purified according to the manufacturer’s protocol (Beckman Coulter; A48705).

#### Retro-orbital injections

50 μL of VLPs (containing 4x10^11^ or 7x10^11^ VLPs) were centrifuged for 10 min at 15,000 *g* to remove debris. The clarified supernatant was diluted to 120 μL in 0.9% NaCl (Fresenius Kabi; 918610) right before injection. 1x10^11^ viral genomes (vg) of total AAV was diluted to 120 μL in 0.9% NaCl (Fresenius Kabi; 918610) right before injection. Anesthesia was induced with 4% isoflurane. Following induction, as measured by unresponsiveness to bilateral toe pinch, the right eye was protruded by gentle pressure on the skin, and an insulin syringe was advanced, with the bevel facing away from the eye, into the retrobulbar sinus where VLP or AAV mix was slowly injected. One drop of Proparacaine Hydrochloride Ophthalmic Solution (Patterson Veterinary; 07-885-9765) was then applied to the eye as an analgesic. Genomic DNA was purified from various tissue using Agencourt DNAdvance kits (Beckman Coulter; A48705) following the manufacturer’s instructions.

#### Histology and staining

Liver tissue was fixed in 4% PFA overnight at 4^°^C. The next day, fixed liver was transferred into 1x PBS with 10 mM glycine to quench free aldehyde for at least 24 h followed by paraffinization at the Rodent Histopathology Core of Harvard Medical School. Liver paraffin block was then cut into 5 μm sections followed by hematoxylin and eosin staining for histopathological examination.

#### Alanine Aminotransferase (ALT) and Aspartate Aminotransferase (AST) assay

Blood was collected 7 days after injection via submandibular bleeding and allowed to clot at room temperature for 1 h. The serum was then separated by centrifugation at 2000 *g* for 15 min and sent to IDEXX Bioanalytics, MA, for analysis.

#### Serum Pcsk9 measurements

To track serum levels of Pcsk9 blood was collected using a submandibular bleed in a serum separator tube. Serum was separated by centrifugation at 2000 *g* for 15 min and stored at -80^o^C. Pcsk9 levels were determined by ELISA using the Mouse Proprotein Convertase 9/PCSK9 Quantikine ELISA Kit (R&D Systems; MPC900) following the manufacturer’s instructions.

#### CIRCLE-seq

Circularization for In vitro Reporting of Cleavage Effects by sequencing (CIRCLE-seq) was performed and analyzed as described previously (Tsai et al., 2017) save for the following modifications: For the Cas9 cleavage step, guide denaturation, incubation, and proteinase K treatment was conducted using the more efficient method described in the CHANGE-seq protocol (Lazzarotto et al., 2020). Specifically, the sgRNA with the guide sequence “GCCCATACCTTGGAGCAACGG” was ordered from Synthego with their standard chemical modifications, 2’O-Methyl for the first three and last three bases, and phosphorothioate bonds between the first three and last two bases. A 5’ “G” nucleotide was included with the 20-nucleotide specific guide sequence to recapitulate the sequence expressed and packaged into VLPs. The sgRNA was diluted to 9 μM in nuclease-free water and re-folded by incubation at 90^°^C for 5 min followed by a slow annealing down to 25^°^C at a ramp rate of 0.1 ^°^C/second. The sgRNA was complexed with Cas9 nuclease (NEB; M0386T) via a 10 min room temperature incubation after mixing 5 μL of 10x Cas9 Nuclease Reaction Buffer provided with the nuclease, 4.5 μL of 1 μM Cas9 nuclease (diluted from the 20μM stock in 1x Cas9 Nuclease Reaction Buffer), and 1.5 μL of 9 μM annealed sgRNA. Circular DNA from mouse N2A cells was added to a total mass of 125 ng and diluted to a final volume of 50 μL. Following 1 h of incubation at 37^°^C, Proteinase K (NEB; P8107S) was diluted 4-fold in water and 5 μL of the diluted mixture was added to the cleavage reaction. Following a 15 min Proteinase K treatment at 37^°^C, DNA was A-tailed, adapter ligated, and USER-treated, and PCR-amplified as described in the CIRCLE-seq protocol (Tsai et al., 2017). Following PCR, samples were loaded on a preparative 1% agarose gel and DNA was extracted between the 300bp and 1kb range to eliminate primer dimers before sequencing on an Illumina MiSeq. Data was processed using the CIRCLE-seq analysis pipeline and aligned to the human genome Hg19 (GRCh37) with parameters: “read_threshold: 4; window_size: 3; mapq_threshold: 50; start_threshold: 1; gap_threshold: 3; mismatch_threshold: 6; merged_analysis: True”.

#### Amplicon sequencing of off-target sites nominated by CIRCLE-seq

We observed in prior work that exhaustively assessed ABE8e off-target sites nominated by CIRCLE-seq that off-target editing efficiency did not track well with the CIRCLE-seq read count (Newby et al., 2021). However, nominated off-target sites where editing was observed shared some striking similarities. Namely, over 90.7% of the 54 off-target sites with validated off-target editing had zero mismatches or one mismatch to the guide in the 9 nucleotides proximal to the PAM. The few sites with more than 1 mismatch in this region were all edited with low efficiency (the bottom half of sites, when ranked by editing efficiency). Based on this knowledge, we chose to assess 14 off-target sites in our CIRCLE-seq list that showed one or fewer mismatches in the 9 nucleotides of the protospacer proximal to the PAM to increase the chance that we sequence a true off-target site (Table S4).

#### Mouse subretinal injection

Mice were anesthetized by intraperitoneal injection of a cocktail consisting of 20 mg/mL ketamine and 1.75 mg/mL xylazine in phosphate-buffered saline at a dose of 0.1 mL per 20 g body weight, and their pupils were dilated with topical administration of 1% tropicamide ophthalmic solution (Akorn; 17478-102-12). Subretinal injections were performed under an ophthalmic surgical microscope (Zeiss). An incision was made through the cornea adjacent to the limbus at the nasal side using a 25-gauge needle. A 34-gauge blunt-end needle (World Precision Instruments; NF34BL-2) connected to an RPE-KIT (World Precision Instruments, no. RPE-KIT) by SilFlex tubing (World Precision Instruments; SILFLEX-2) was inserted through the corneal incision while avoiding the lens and advanced through the retina. Each mouse was injected with 1 μL of experimental reagent (lentivirus or eVLPs) per eye. Lentivirus titer was >1x10^9^ TU/mL as measured by the QuickTiter™ Lentivirus Titer Kit (Cell Biolabs; VPK-107-5). BE-eVLPs were normalized to a titer of 4x10^10^ eVLPs/μL, corresponding to an encapsulated BE protein content of 3 pmol/μL. After injections, pupils were hydrated with the application of GenTeal Severe Lubricant Eye Gel (0.3% Hypromellose, Alcon) and kept for recovery.

#### RPE dissociation and genomic DNA and RNA preparation

Under a light microscope, mouse eyes were dissected to separate the posterior eyecup (containing RPE, choroid and sclera) from the retina and anterior segments. Each posterior eyecup was immediately immersed in 350 μl of RLT Plus tissue lysis buffer provided with AllPrep DNA/RNA Mini Kit (Qiagen; 80284). After 1 min incubation, RPE cells were detached in the lysis buffer from the posterior eyecup by gentle pipetting, followed by a removal of the remaining posterior eyecup. The lysis buffer containing RPE cells was further processed for DNA and RNA extraction using the AllPrep DNA/RNA Mini Kit protocol. The final DNA and RNA were eluted in 30 μL and 15 μL water, respectively. cDNA synthesis was performed using the SuperScript™ III First-Strand Synthesis SuperMix (Thermo Fisher; 18080400).

#### Western blot analysis of mouse RPE tissue extracts

To prepare the protein lysate from the mouse RPE tissue, the dissected mouse eyecup, consisting of RPE, choroid, and sclera, was transferred to a microcentrifuge tube containing 30 μL of RIPA buffer with protease inhibitors and homogenized with a motor tissue grinder (Fisher Scientific; K749540-0000) and centrifuged for 30 min at 20,000 *g* at 4°C. The resulting supernatant was pre-cleared with Dynabeads Protein G (Thermo Fisher; 10003D) to remove contaminants from blood prior to gel loading. Twenty μL of RPE lysates pre-mixed with NuPAGE LDS Sample Buffer (Thermo Fisher; NP0007) and NuPAGE Sample Reducing Agent (Thermo Fisher; NP0004) was loaded into each well of a NuPAGE 4-12% Bis-Tris gel (Thermo Fisher; NP0321BOX), separated for 1 h at 130 V and transferred onto a PVDF membrane (Millipore; IPVH00010). After 1 h blocking in 5% (w/v) non-fat milk in PBS containing 0.1% (v/v) Tween-20 (PBS-T), the membrane was incubated with primary antibody, mouse anti-RPE65 monoclonal antibody (1:1,000; in-house production) (Golczak et al., 2010), diluted in 1% (w/v) non-fat milk in PBS-T overnight at 4°C. After overnight incubation, membranes were washed three times with PBS-T for 5 min each and then incubated with goat anti-mouse IgG-HRP antibody (1:5,000; Cell Signaling Technology; 7076S) for 1 h at room temperature. After washing the membrane three times with PBS-T for 5 min each, protein bands were visualized after exposure to SuperSignal West Pico Chemiluminescent substrate (Thermo Fisher; 34580). Membranes were stripped and reprobed for ABE and β-actin expression using mouse anti-Cas9 monoclonal antibody (1:1,000; Invitrogen; MA523519) and rabbit anti-β-actin polyclonal antibody (1:1,000; Cell Signaling Technology; 4970S), following the same protocol. Corresponding secondary antibodies were goat anti-mouse IgG-HRP antibody (1:5,000; Cell Signaling Technology; 7076S) and goat anti-rabbit IgG-HRP antibody (1:5,000; Cell Signaling Technology; 7074S).

#### Electroretinography

Prior to recording, mice were dark adapted for 24 h overnight. Under a safety light, mice were anesthetized by intraperitoneal injection of a cocktail consisting of 20 mg/mL ketamine and 1.75 mg/mL xylazine in phosphate-buffered saline at a dose of 0.1 mL per 20 g body weight, and their pupils were dilated with topical administration of 1% tropicamide ophthalmic solution (Akorn; 17478-102-12) followed by 2.5% hypromellose (Akorn; 9050-1) for hydration. The mouse was placed on a heated Diagnosys Celeris rodent ERG device (Diagnosys LCC). Ocular electrodes were placed on the corneas, and the reference electrode was positioned subdermally between the ears. The eyes were stimulated with a green light (peak emission 544 nm, bandwidth ∼160 nm) stimulus of -0.3 log (cd·s/m^2^). The responses for 10 stimuli with an inter-stimulus interval of 10 s were averaged together, and the a- and b-wave amplitudes were acquired from the averaged ERG waveform. The ERGs were recorded with the Celeris rodent electrophysiology system (Diagnosys LLC) and analyzed with Espion V6 software (Diagnosys LLC).

### Quantification and statistical analysis

Data are presented as mean and standard error of the mean (SEM). No statistical methods were used to predetermine sample size. Statistical analysis was performed using GraphPad Prism software. Sample size and the statistical tests used are described in the figure legends.

## Data Availability

The sequencing data generated during this study are available at the NCBI Sequence Read Archive database under PRJNA768458. The code used for data processing and analysis are available at https://github.com/pinellolab/CRISPResso2.

## Supplemental information

Table S1. Protein sequences of BE-eVLP constructs, related to Figures 1, 2, and 3

Table S2. Sequences of protospacers, primers, and amplicons used for high-throughput sequencing analyses, related to Figures 1, 2, 3, 4, 5, 6, and 7

Table S3. Sequences of primers used for qPCR or RT-qPCR analyses, related to Figure 3

Table S4. Sequences of Pcsk9 off-target sites identified by CIRCLE-seq, related to Figure 6
